# Molecular tools for GABA_A_ receptors: High affinity ligands for β1-containing subtypes

**DOI:** 10.1038/s41598-017-05757-4

**Published:** 2017-07-18

**Authors:** Xenia Simeone, David C. B. Siebert, Konstantina Bampali, Zdravko Varagic, Marco Treven, Sabah Rehman, Jakob Pyszkowski, Raphael Holzinger, Friederike Steudle, Petra Scholze, Marko D. Mihovilovic, Michael Schnürch, Margot Ernst

**Affiliations:** 1 Department of Molecular Neurosciences, Center for Brain Research, Medical University Vienna, Spitalgasse 4, 1090 Vienna, Austria; 2 Institute of Applied Synthetic Chemistry, TU Wien, Getreidemarkt 9/163, 1060 Vienna, Austria; 3 Department of Pathobiology of the Nervous System, Center for Brain Research, Medical University of Vienna, Spitalgasse 4, 1090 Vienna, Austria

## Abstract

γ-Aminobutyric acid type A (GABA_A_) receptors are pentameric GABA-gated chloride channels that are, in mammalians, drawn from a repertoire of 19 different genes, namely α1-6, β1-3, γ1-3, δ, ε, θ, π and ρ1-3. The existence of this wide variety of subunits as well as their diverse assembly into different subunit compositions result in miscellaneous receptor subtypes. In combination with the large number of known and putative allosteric binding sites, this leads to a highly complex pharmacology. Recently, a novel binding site at extracellular α+/β− interfaces was described as the site of modulatory action of several pyrazoloquinolinones. In this study we report a highly potent ligand from this class of compounds with pronounced β1-selectivity that mainly lacks α-subunit selectivity. It constitutes the most potent β1-selective positive allosteric modulatory ligand with known binding site. In addition, a proof of concept pyrazoloquinolinone ligand lacking the additional high affinity interaction with the benzodiazepine binding site is presented. Ultimately, such ligands can be used as invaluable molecular tools for the detection of β1-containing receptor subtypes and the investigation of their abundance and distribution.

## Introduction

GABA_A_ receptors are pentameric ligand-gated ion channels that can be opened by GABA and alternative agonists, as well as modulated by multiple endogenous or exogenous allosteric ligands, some of which have high clinical importance^1^. In the nervous system GABA_A_ receptors are, among others, targets of certain sleeping aids, general anesthetics and antiepileptic medications. High affinity ligands of the benzodiazepine binding site of these receptors are also used as versatile CNS imaging tools^2^. Specific receptor subtypes also occur in diverse peripheral tissues where their function is largely unknown^3, 4^.

A total of 19 genes encode, in mammalian species, GABA_A_ receptor subunits (α1-6, β1-3, γ1-3, δ, ε, θ, π and ρ1-3)^5^. Specific subunits assemble into homo- or hetero- pentameric arrangements, whereby a given pentamer with defined subunit composition and arrangement is referred to as receptor subtype. The receptor subtype composed of α1, β3 and γ2 subunits was shown to be arranged as β3-α1-γ2-β3-α1^6^, where each subunit interface by definition has a principal (plus) and a complementary (minus) side^7^. The total number of pentameric arrangements that exist in mammalian species is still unknown^5^, but given the repertoire of 19 subunits, it could be large.

The conserved cys-loop receptor structure harbors a large number of binding sites, including those for the generic agonist GABA, for channel blockers such as picrotoxin, and for a wide range of allosteric modulators^8^. Each binding sites’ ligand preferences are determined by the subunits that contribute to it. The ion channel pore is formed by the five transmembrane domain two segments (TM2)^9, 10^. Agonist sites are at extracellular interfaces between specific subunits such as the bicuculline insensitive ρ+/ρ− and the bicuculline sensitive β+/α− sites^11^. Allosteric sites have been described at interfaces and in other locations in the extracellular and transmembrane domains^8^.

Together, the staggering variety of receptor subtypes and the large number of binding sites on each subtype results in a highly complex pharmacology^1^. Specific high affinity ligands of GABA_A_ receptor subtypes are invaluable tools to study their abundance and distribution in tissues and to detect them in living organisms. Unselective high affinity ligands that can be employed to detect large pools of GABA_A_ receptors exist for specific applications, such as autoradiography and radioligand binding studies^12^. In contrast, only very few tool compounds exist that display specific high affinity binding at individual subtypes. Selective molecular tools do exist for the widely expressed γ2 subunit containing receptors. A number of high affinity benzodiazepine site ligands have been developed that facilitate their selective detection in biological samples and *in vivo* ^2, 12^. These ligands bind to the high affinity benzodiazepine binding site that is formed by a principal α subunit (α1, α2, α3 or α5) together with a complementary γ2 subunit^13^. Among them, ligands that bind with higher affinity to α5+/γ2− have been identified, such as Ro 15-4513, which is used as α5-specific PET ligand to detect the receptor subtypes containing α5+/γ2− binding sites in humans^14, 15^.

In contrast, no high affinity ligands exist that are selective for receptors containing a specific β isoform. Fragrant dioxane derivatives (FDDs) have been described as a novel structural class of GABA_A_ receptor positive modulators with β1-subunit selectivity and have been proven useful for functional studies^16^. However, the relatively low micromolar potency prevents their use as radioligands^16^. Furthermore, salicylidene salicylhydrazide (SCS) has been described as potent partial and selective antagonist of β1-containing receptors^17^ and has been used successfully for the functional identification of β1-containing receptors. Nevertheless, it has not been developed into a tool for binding studies so far. For both FDDs and SCS binding sites are unknown. In contrast, a number of modulators for the GABA_A_ receptor with enhanced selectivity for the β2/β3 subunits over the β1 subunit have been reported, e.g. etomidate, loreclezole and a valerenic acid derivative^18–20^. Studies in transgenic animals have shown that compounds that target individual β isoforms selectively would be highly useful, as different effects of sedative and anesthetic compounds could be separated^21^.

**Figure 1:**
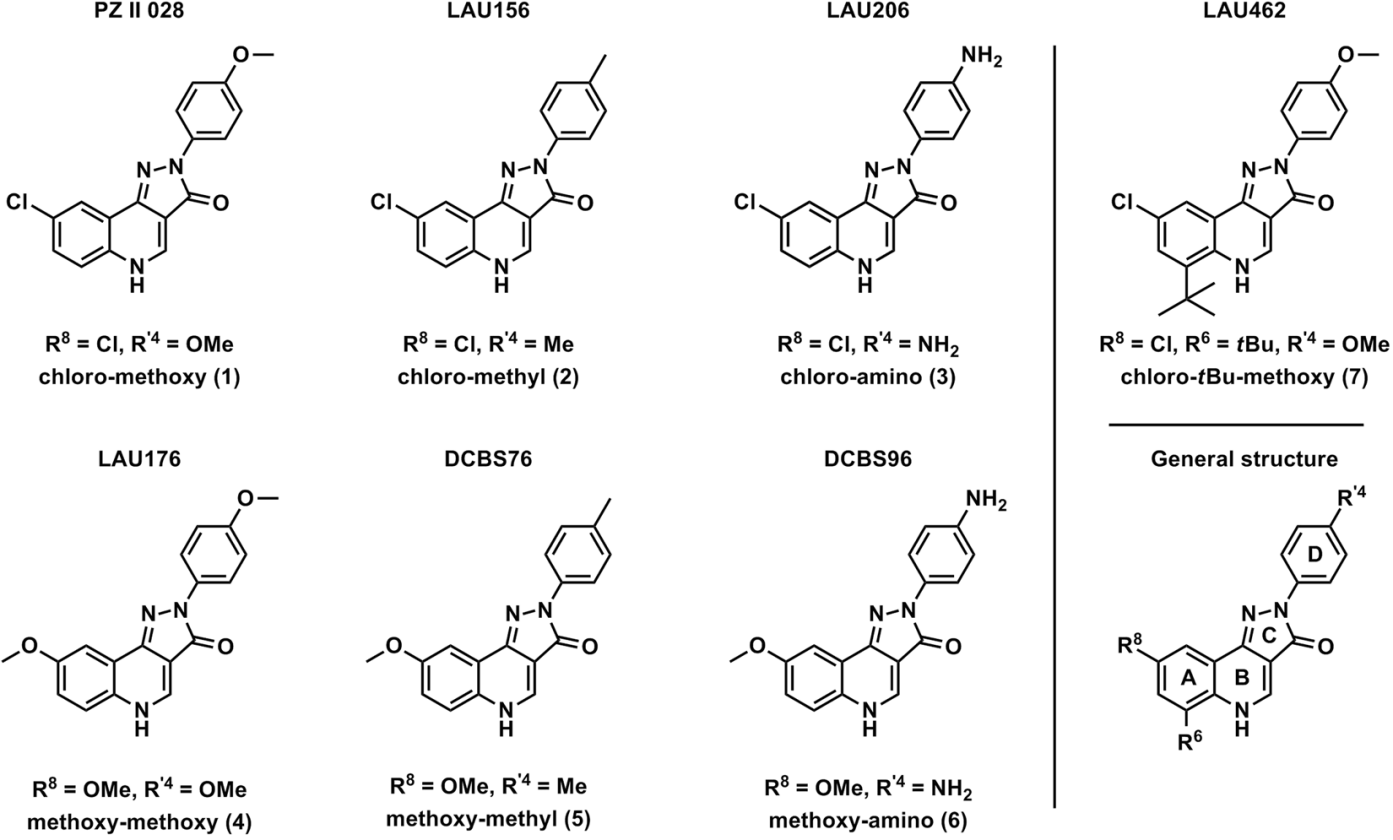
Chemical structures of the ligands (**1**–**7**) employed in this study. The letters “A, B, C and D” refer to the different rings in the scaffold. The position and numbering of the residues (R^6^, R^8^ and R′^4^) are depicted in the general structure (bottom right corner). The nomenclature “chloro-methoxy” or “methoxy-methoxy” respectively describes first the residue in position R^8^ and then the residue in position R′^4^. For compound **7** residues in position R^8^-R^6^-R′^4^ are indicated.

We have recently described allosteric modulation of diverse GABA_A_ receptors by several pyrazoloquinolinones (PQs) that use a binding site at extracellular α+/β− interfaces^22–25^. Since all six α isoforms and all three β isoforms contribute unique amino acid residues to that binding site, it should be possible to identify highly selective ligands for any αk+/βl− (k = 1–6, l = 1–3) combination. We have previously studied 32 pyrazoloquinolinones and pyrazolopyridinones at the α1+/β3− binding site^23^, and 16 of those were investigated for possible α subtype selectivity^22^. Among the compounds that up until now were only studied as ligands of the α1+/β3− binding site, we selected for this follow up study three analogues which modulated α1β3 receptors with efficacy higher than 300%^23^ and three analogues thereof (see Fig. 1). Here possible potency selectivity for either α isoforms, or β isoforms was investigated.

We identified, and present here, a highly potent ligand (**1**) of the α+/β1− sites featuring an EC_50_ of 130 nM at α1+/β1− interfaces. This ligand is also a benzodiazepine site ligand, and thus not a selective tool for α1+/β1− interfaces. Consequently, we also generated an analogue (**7**) that lacks benzodiazepine site interaction while largely retaining the desired activity at the homologous α1+/β1− interface site. These studies pave the way towards high affinity molecular tools for the selective detection of receptor subtypes that contain specific αk+/βl− (any of k = 1–6, l = 1–3) interfaces.

## Results

### Mini library of compounds aimed at studying potency driving ligand features

In our previous work we identified compounds **1**–**3** to be efficacious modulators of the extracellular α1+/β3− interface site^23^, and thus selected these for a follow up study to investigate potential potency preferences for any subtype. Due to the strong impact of the R^8^ substituent on compound efficacy^23^, we added three more analogues (compounds **4**–**6**) with another residue in this position. Ligand **7** (see Fig. 1) was added later to confirm the observation that bulk in R^6^ interferes with the unwanted benzodiazepine site affinity while, at least for some ligands, retaining modulatory action at the extracellular α1+/β3− interface site^23^.

### Compound 1 exerts very similar effects in α1β3, α1β3γ2 and α1β3δ receptors

**Figure 2:**
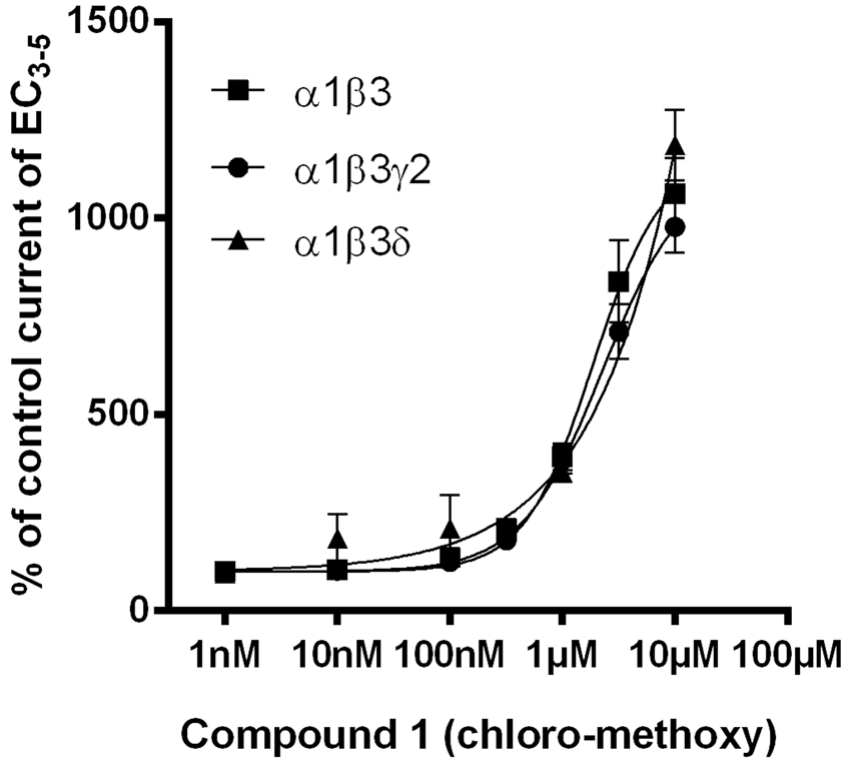
Compound **1** modulates GABA-evoked currents from α1β3, α1β3γ2 and α1β3δ similarly. Concentration-dependent modulation of GABA EC_3–5_ current at α1β3, α1β3γ2 and α1β3δ. Data represent means ± SEM (n = 3–8). α1β3 and α1β3γ2 data are identical with those published previously^22^.

As we have described previously, many R^8^ and R′^4^ di-substituted pyrazoloquinolinones not only interact with the α+/β− interfaces, but also bind with very high affinity to α+/γ2− interfaces (benzodiazepine binding sites)^22, 23, 26^. For a library screen, binary αβ receptors offer the advantages that they lack the high affinity benzodiazepine binding site, and express robustly, quickly and consistently in the *Xenopus laevis* oocyte. To clarify whether the use of binary receptors gives satisfactory results, we carefully investigated the modulatory effects of compound **1** in α1β3, α1β3γ2 (diazepam sensitive, see methods) and α1β3δ (DS2 sensitive, see methods) expressing oocytes as shown in Fig. 2.

Since the modulatory effects that are exerted from the α+/β− interface are nearly unaffected by the presence of a γ2 or a δ subunit, we proceeded to screen our mini library in binary receptors. At the experimental conditions used in this study, the α1βl (l = 1, 2, 3) receptors formed in the oocyte are thought to be of α1(2)βl(3) stoichiometry^27^.

### Potency selectivity for β1-containing receptors

**Figure 3:**
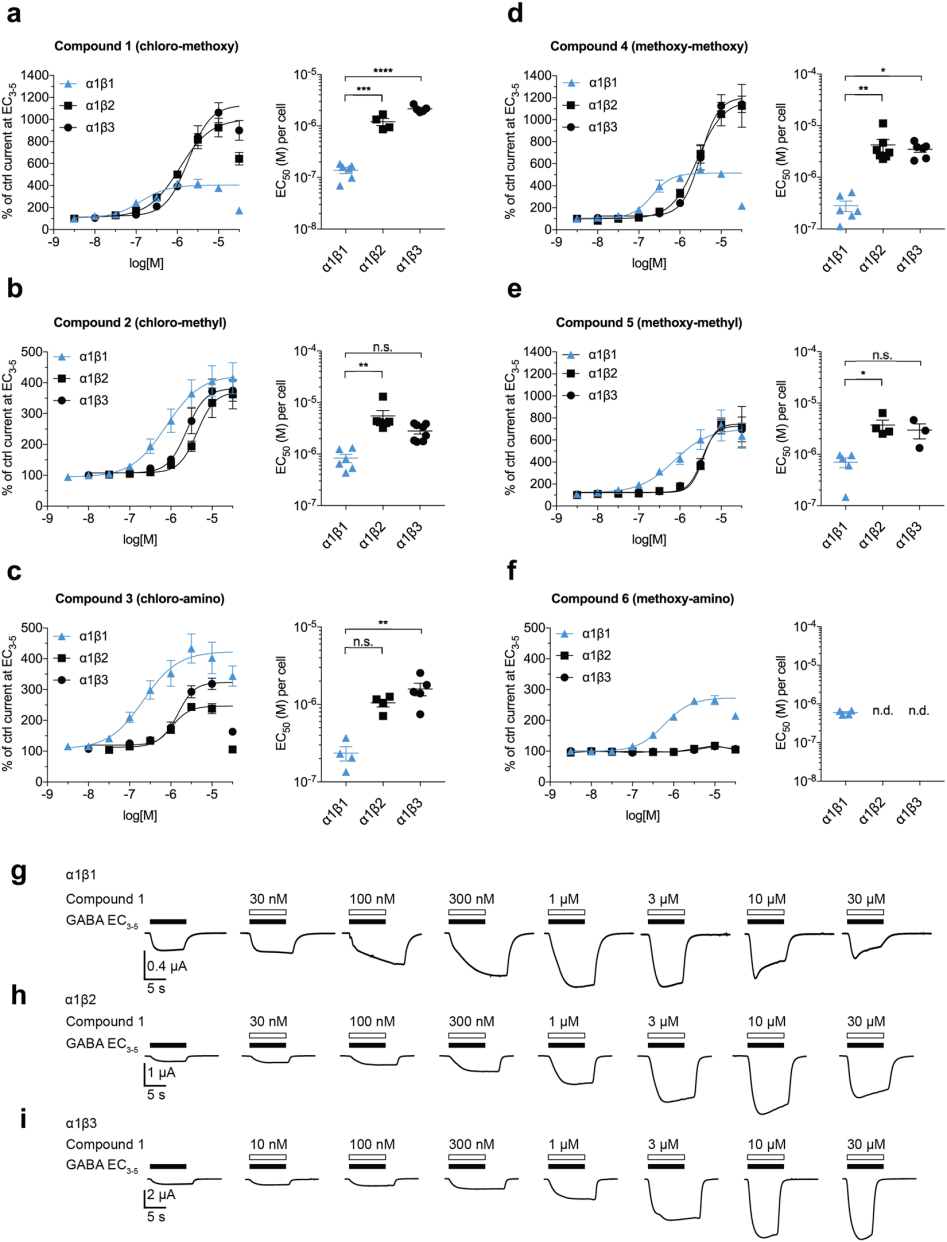
Compounds **1**–**6** show potency selectivity for β1-containing receptors. Dose-response data of compounds **1**–**6** at α1β1, α1β2 and α1β3 subunit combinations; (**a**–**c**) Left, aggregate dose-response curves of R^8^ = chloro compounds **1**–**3** co-applied with GABA EC_3–5_. Right, EC_50_ values obtained by fitting data of each cell individually; (**d**–**f**) Left, aggregate dose-response curves of R^8^ = methoxy compounds **4**–**6** co-applied with GABA EC_3–5_. Right, EC_50_ values obtained by fitting data of each cell individually. Highest potency was consistently observed at α1β1 receptors. Compound **6** (**f**) lacked efficacy at α1β2 and α1β3, therefore EC_50_ values could not be obtained. In those instances where high compound concentrations elicited substantial desensitization (see panels **a**, **c**, **d**, **f** and sample traces in (**g,h**)), the highest compound concentration was excluded from the fit. Statistically significant differences were assessed by one-way ANOVA with Tukey’s multiple comparison test; *p < 0.05, ***p < 0.01, ****p < 0.001, *****p < 0.0001, n.s. = not significant, n.d. = not determined. n = 3–8. (**g**–**i**) Sample traces obtained with compound **1**. Note the desensitization in α1β1 (**g**) at 10 µM and 30 µM, increasingly limiting maximum current amplitudes. Tabulated data corresponding to panels **a**–f are provided in Supplementary Tables S1–S6. Additional sample traces are provided in Supplementary Fig. S7.

Next, we searched for β selectivity in α1βl (l = 1, 2, 3) receptors (see Fig. 3a–f). First, compounds **1**–**6** were investigated without GABA using α1βl (l = 1, 2, 3) expressing oocytes. None of the compounds displayed any GABA independent effects at 10 and 30 µM. Compound modulatory effects were then investigated at low GABA concentrations (corresponding to EC_3–5_), where all compounds showed to be positive modulators, and all of them displayed higher potency in α1β1 receptors than in α1β2 or α1β3 receptors (see Fig. 3). The most potent ligand in α1β1 is compound **1** (EC_50_:130 nM). Moreover, compounds **3** and **4** also display high potencies (~200 nM) for α1β1. Potency differences between α1β1 and α1β2 are statistically significant for compounds **1**, **2**, **4** and **5** (p < 0.001, p < 0.01, p < 0.01 and p < 0.05, respectively). Furthermore, compounds **1**, **3** and **4** (p < 0.0001, p < 0.01, and p < 0.05, respectively) also show statistically significant potency differences between α1β1 and α1β3.

All compounds have approximately the same efficacy in α1β1 and enhance the GABA EC_3–5_ currents in this subtype up to ~400%. On the other hand, the efficacy in β2- and β3-containing receptors varies widely: **1** and **4** have much higher efficacy in α1β2 and α1β3 compared to α1β1, **2** and **5** modulate all three receptors to the same degree, while **3** and **6** display reduced efficacy in α1β2 and α1β3 compared to α1β1 (see Fig. 3).

We observed that all six ligands influenced receptor kinetics in a way such that at low compound concentrations, the current rise was delayed compared to the reference GABA trace (see Fig. 3g and Supplementary Fig. S7 panel f). Interestingly, compounds **1** and **3**, as well as **4** and **6** also accelerate current decay at high concentrations (see panel g in Fig. 3 and Supplementary Fig. S7), while **2** and **5** do not. A similar phenomenon has been observed and reported previously for an unrelated allosteric modulator^28^. We explain the apparent drop in efficacy at high concentrations of compounds **1** and **3** by the accelerated current decay. At very high concentrations, the current decay is so fast that the peak amplitude of the initial current enhancement drops (see Fig. 3g). Thus, to obtain compound EC_50_ values, only the data points that fall on the sigmoidal phase of the curve were utilized (see Supplementary Tables S1–S6).

The Hill slopes of the compound dose-response curves range from 1 to 3 (see Supplementary Tables S1–S6). This is consistent with the recently proposed view that some pyrazoloquinolinones may have additional binding sites in the transmembrane domain of certain specific subunit combinations^29^.

### Mutational analysis supports the main site of action to be at the extracellular minus side of the β subunit

As additional binding sites for pyrazoloquinolinones have been proposed^29^, we aimed to investigate the molecular determinants which lead to the potency preference of our test ligands for the β1 isoform. We compared the different extracellular minus sides utilizing homology models based on the recently published β3-homopentameric crystal structure^30^. These models (see Supplementary Fig. S8) indicate that the amino acids corresponding to β1R41 and β3N41 (numbering according to mature rat protein without signal peptide) on segment (or “loop”) G, which is structurally a strand within a beta-pleated sheet, are in the variable position most central in the pocket. These are in close interaction with the predicted ligand occupied space (see Supplementary Fig. S8). In the benzodiazepine binding site, the homologous sub-domain has been shown to impact on ligand binding^31^. To test the influence of this amino acid on potency and efficacy of our ligands, two “conversion” mutants were generated. By the point mutations β3N41R and β1R41N the variable amino acid on segment G was exchanged between these two isoforms, leading to two engineered subunits. These presumably display properties mostly derived from the parent subunit, but locally changed ligand interactions.

The binary α1β1R41N receptor displayed variable and often large holding currents which are indicative of spontaneous channel activity. This phenomenon has been described for several point mutations that also displayed spontaneous currents^32, 33^. On the other hand, the binary α1β3N41R receptor behaved similarly as the wild type α1βl (l = 1, 2, 3) receptors. The GABA dose-response curves of both mutants are slightly left shifted (EC_50_ ~2 µM) compared to the ones of the wild type α1β1 or α1β3 receptors (see Supplementary Fig. S9 and Supplementary Table S10). Maximum GABA currents are similar as in the wild type receptors, and the Hill coefficients are ~1.2 for both mutated receptors (see Supplementary Table S10).

**Figure 4:**
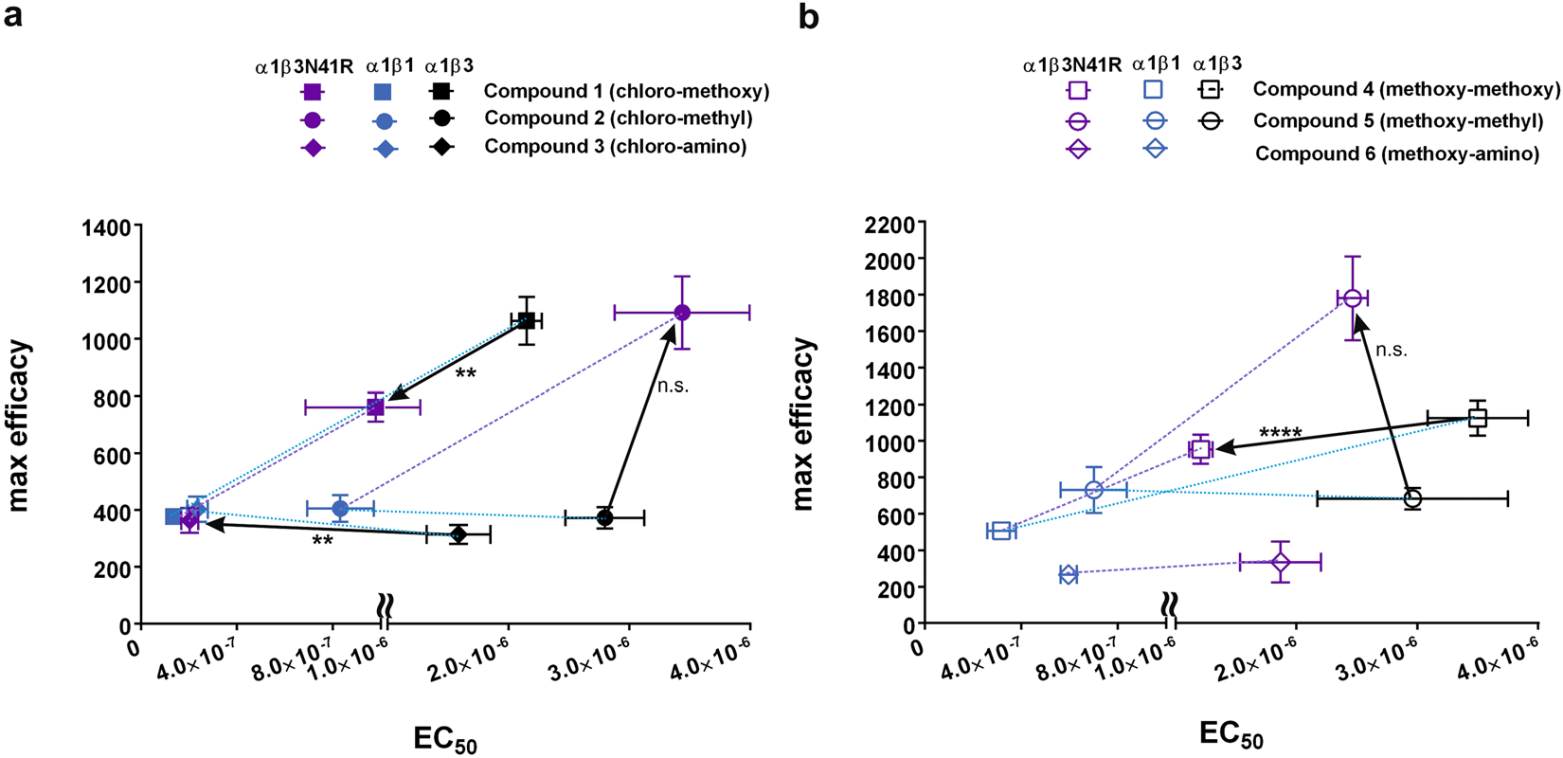
Comparison of EC_50_ and maximum efficacy among α1β1, α1β3 and α1β3N41R. (**a,b**) The plots show the mean EC_50_ on the x-axis (note that the axis is broken to accommodate the range) and the mean maximum efficacy at 10 μM (% of control current at EC_3–5_) on the y-axis (note the different scales on the two panels) of compounds **1**–**6**. The difference between wild type α1β3 and α1β3N41R is indicated with a black arrow, statistically significant EC_50_ differences are indicated. The potency differences between α1β3 and α1β3N41R for compounds **1**, **3** and **4** are statistically significant (**,**,****, respectively). Arrows pointing to the left show a decrease of the EC_50_ value between wild type and mutated receptors, which corresponds to an increase in potency. Simultaneously, changes in efficacy can be seen (arrows with upward or downward component indicating increase or decrease in maximum efficacy, respectively). The values obtained with wild type α1β3 and α1β1 receptors are connected with a blue dotted line. The dotted purple line visualizes the difference between α1β1 and α1β3N41R. The EC_50_ values for the mutated receptors are 0.98 µM, 3.44 µM, 0.2 µM, 1.2 µM, 2.47 µM and 1.87 µM for compounds **1**–**6**, respectively. EC_50_ values were calculated for each individual experiment and are presented as mean ± SEM. Statistically significant differences were assessed by one-way ANOVA with Tukey’s multiple comparison test. Note that the EC_50_ value of compound **6** in α1β3 receptors is not depicted, since this compound has nearly no efficacy in this receptor subtype. Bars indicate mean ± SEM, n = 3–8. The dose-response curves of compounds **1**–**6** in α1β3N41R receptors are depicted in Supplementary Fig. S12.

Next, the modulatory effects of the compounds were examined in both mutated receptors. Interestingly, the positive modulatory effects are completely abolished for three ligands and dramatically reduced for compounds **1** and **4**, whereas compound **2** is reducing GABA currents in the α1β1R41N receptor (see Supplementary Table S11). In contrast, the α1β3N41R combination displayed modulatory responses to all ligands. We observed significant changes in potency compared to the wild type (parent) α1β3 receptor for three ligands (see Fig. 4). For one ligand (**6**) potency in the wild type could not be determined due to the very low efficacy – but interestingly the loop G mutation induced β1-like efficacy in this case (see Supplementary Fig. S12). For two additional ligands, we noted an increase in efficacy as a result of the mutant (see Fig. 4).

Interestingly, the substituent in R′^4^ seems to determine how the ligand interacts with the mutated β subunit. Both compounds with a methoxy substituent in R′^4^ show a left shift with a resulting potency in the mutant receptor that is intermediate between the values of the two wild type receptors α1β1 and α1β3 (see Fig. 4, Supplementary Fig. S12). For compounds bearing R′^4^ = methyl we observed a strong enhancement of efficacy in the α1β3N41R receptor (see Fig. 4, Supplementary Fig. S12), with no significant change in potency. For the R′^4^ = amino substituted compound **3**, the mutation led to a marked left shift such that potency for α1β3N41R and for α1β1 are identical (complete conversion, see Fig. 4 and Supplementary Fig. S12). Overall, these observations demonstrate that both potency and efficacy of the PQ compounds are differentially determined in part by the amino acid in position 41 of the minus side segment G in α1β1 and α1β3 receptors and thus strongly support the notion that the modulatory effects are mainly elicited by the tested ligands at the extracellular α+/β− interface^23^.

### The investigated compounds show limited α selectivity

Each R^8^ = chloro compound was more potent in the α1β1 receptor compared to their respective methoxy analogues, thus, we followed up in more detail on compounds **1, 2** and **3**. Compound **1** already has been investigated in 22 receptor subtypes, namely in αkβ3 (k = 1, 2, 3, 5) and αkβlγ2 (k = 1–6, l = 1–3), and displayed nearly no potency differences among αkβlγ2 (k = 1–6, l = 1–3) or αkβ3 (k = 1, 2, 3, 5) receptors (see Supplementary Table S13), while displaying pronounced functional preference for α6-containing receptors^22^. Thus, we investigated a possible α subtype selectivity of compounds **2** and **3**. For αkβ3γ2 (k = 1–6) combinations the expression protocols are well established and all combinations express reasonably well showing consistent responses to diazepam for αkβ3γ2 (k = 1, 2, 3, 5)^22^. In contrast, for β1-containing combinations, this is not the case and some combinations proved to be difficult to express and characterize. Thus, in order to study the impact of the α isoforms, we utilized the β3 subunit throughout. Table 1 shows the EC_50_ and pEC_50_ values obtained for the six αkβ3γ2 (k = 1–6) subunit combinations for compounds **2** and **3**.

**Table 1:** Impact of α isoform on potency of compound **2** and **3**.

	compound 2 (chloro-methyl)				compound 3 (chloro-amino)			
	EC_50_ [µM]	pEC_50_	SEM	(n)	EC_50_ [µM]	pEC_50_	SEM	(n)
α1β3γ2	4.4	5.4	0.3	(3)	1.2	5.9	0.1	(3)
α2β3γ2	5.5	5.3	0.2	(9)	n.d.*	n.d.*	n.d.*	(6)
α3β3γ2	7.2	5.1	0.7	(3)	1.2	5.9	0.9	(4)
α4β3γ2	13.4	4.9	0.6	(3)	n.d.*	n.d.*	n.d.*	(7)
α5β3γ2	6.4	5.2	0.4	(3)	n.d.*	n.d.*	n.d.*	(6)
α6β3γ2	>5	n.d.*	n.d.*	(8)	2.3	5.6	0.7	(5)

EC_50_, pEC_50_ and SEM of R^8^ = Cl compound **2** (LAU156) and **3** (LAU206) are shown. Supplementary Tables S14 and S15 show tabulated dose-response data. Compound **2** modulates all receptors, EC_50_ ranges from 4 µM to 13 µM, where in α6β3γ2 the EC_50_ value could not be obtained as saturation was not reached. Compound **3** has moderate modulatory effects in α1- and α3β3γ2 receptors with an EC_50_ at ~1 µM and in α6β3γ2 with an EC_50_ of ~2 µM. Due to the extremely low efficacy in α2-, α4- and α5β3γ2, these EC_50_ values could not be obtained; (n.d. = not determined).

Compound **2** modulates all six αkβ3γ2 (k = 1–6) subtypes with EC_50_ values in the range ~5 to ~15 µM (see Table 1), and thus without any marked potency selectivity for any of the six α isoforms. The maximum efficacies were also not indicative of any efficacy-selective effect, ranging from ~200% in the α5-containing receptor subtype to ~600% in the α1- containing subtype, with the exception of the α6β3γ2 subtype which displayed higher efficacy (>1000% modulation at 10 µM, see Supplementary Table S14).

Compound **3** exerts modulatory effects at α1- and α3β3γ2 receptors up to ~200% with an EC_50_ of ~1 µM (see Supplementary Table S15). The α6β3γ2 subtype once again was modulated with the highest efficacy in comparison. Due to the low efficacies in the αk-containing (k = 2, 4, 5) receptors, EC_50_ values could not be determined in these receptors, but can be estimated to be in the micromolar, >10 µM, range.

Four of the α isoforms, namely α1, α2, α3 and α5 also produce binary αkβ3 receptors with robust GABA currents, while α4β3 and α6β3 receptors feature very small GABA currents^34^. We compared αkβ3 (k = 1, 2, 3 and 5 that are diazepam insensitive) with αkβ3γ2 (k = 1, 2, 3 and 5 that are diazepam sensitive) receptors and once again found no impact of the γ2 subunit on potency^23, 25^. As expected, potencies (EC_50_) were found to be very similar in the binary receptors as in the corresponding αkβ3γ2 (k = 1, 2, 3 and 5) receptors (see Supplementary Tables S14–S17). Only in one instance a drop in efficacy due to the presence of the γ2 subunit was seen, namely for compound **3** effects in α2β3 compared to α2β3γ2 (see Supplementary Tables S15 and S17).

Overall, the data demonstrate the impact of α isoforms on potency of these ligands is rather limited, and that the presence of the benzodiazepine binding sites formed by α1, α2, α3 or α5 subunits is also silent. Together with the results in the α1βl (l = 1, 2, 3) combinations (see Fig. 3), we identified compound **1** as the most potent ligand for the extracellular α1+/β1− site and thus followed up on compound **1** in more detail.

### The δ and the γ1 subunits have no impact on compound 1 potency for the α1+/β1− site

**Figure 5:**
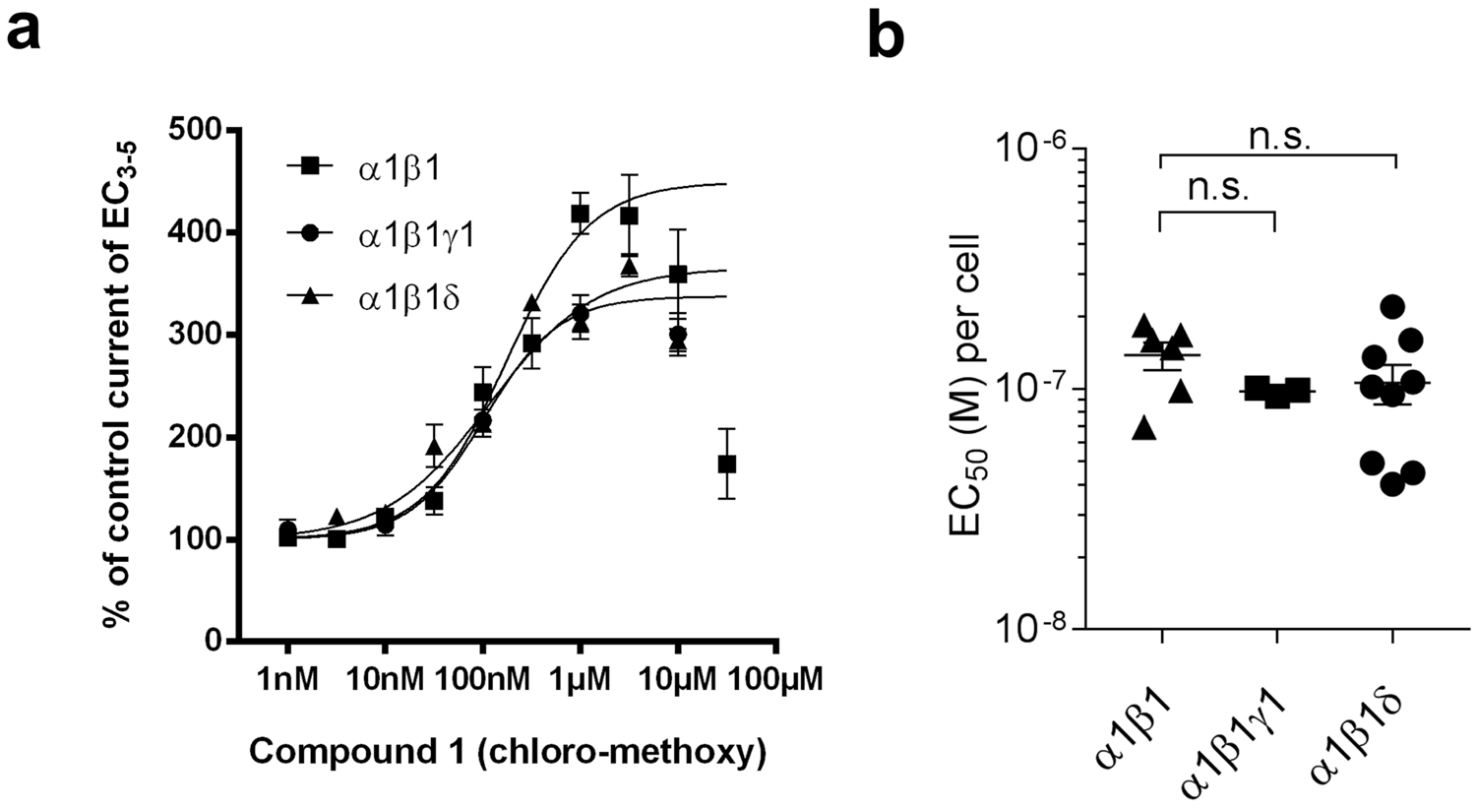
Compound **1** modulates GABA-evoked currents in α1β1, α1β1γ1 and α1β1δ receptors with similar potencies. (**a**) Concentration-dependent modulation of GABA EC_3–5_ current at α1β1, α1β1γ1 and α1β1δ. Data represent means ± SEM (n = 3–10). (**b**) EC_50_ values were calculated for each individual experiment and are presented as mean ± SEM. One-Way ANOVA was used for multiple comparisons followed by a *Tukey post hoc* test and showed no significant differences between the mean EC_50_ values for each subtype.

For compound **1** the previously published data indicates that there is very little influence of the γ2 subunit on the modulatory effect^22, 23^ in spite of the very high potency of this compound for the diazepam sensitive benzodiazepine sites^26^ of αkβ3γ2 receptors (k = 1, 2, 3 and 5). Here we investigated the question whether the more potent interaction with the α1+/β1− site is also not influenced by the presence of a third subunit. We obtained consistent GABA responses, as well as consistent modulation by triazolam for α1β1γ1 receptors (triazolam sensitive, see methods and Supplementary Table S18) while the incorporation of γ2 seemed to be more variable. Similarly, the α1β1δ combination (DS2 sensitive, see methods) also proved to be well behaved (see Supplementary Table S18). Figure 5 shows that the potency and efficacy of compound **1** are not changed by the presence of either the γ1 or the δ subunit.

### A derivative of compound 1 that lacks affinity for the benzodiazepine binding site also modulates α1β1-containing receptors

Since many R^8^ and R′^4^ substituted pyrazoloquinolinones not only interact with the α+/β− interfaces, but are very high affinity ligands at αk+/γ2- interfaces (i.e. benzodiazepine site ligands)^22, 23, 26^, we examined the affinity of our six test ligands for the α1+/γ2- site with flunitrazepam displacement assays using cerebellar membrane preparations from rat brains. The data indicate that all six ligands from the mini library are high affinity binders at the major α1+/γ2- benzodiazepine binding site (see Table 2).

**Table 2:** K_i_ values of compounds **1**–**7** determined by displacement of [^3^H]flunitrazepam binding to rat cerebellar membranes (mean ± SEM, n = 3–4).

R^6^	R^4^	R^8^ = Cl		(n)	R^8^ = OCH_3_		(n)
		cpd	K_i_ [nM]		cpd	K_i_ [nM]	
H	OMe	1	0.06 ± 0.02	(3)	4	0.07 ± 0.007	(4)
H	Me	2	0.05 ± 0.001	(3)	5	0.05 ± 0.002	(3)
H	NH_2_	3	0.12 ± 0.03	(3)	6	1.00 ± 0.08	(3)
*t*Bu	OMe	7	n.d. >100 μM	(3)	—	—	—

We have reported previously an R^6^ substituted pyrazoloquinolinone with dramatically reduced benzodiazepine site affinity and robust α+/β− modulatory effects^23^. Thus, here we investigated the possibility that an analogous derivatization of compound **1** may result in similar ligand properties.

**Figure 6:**
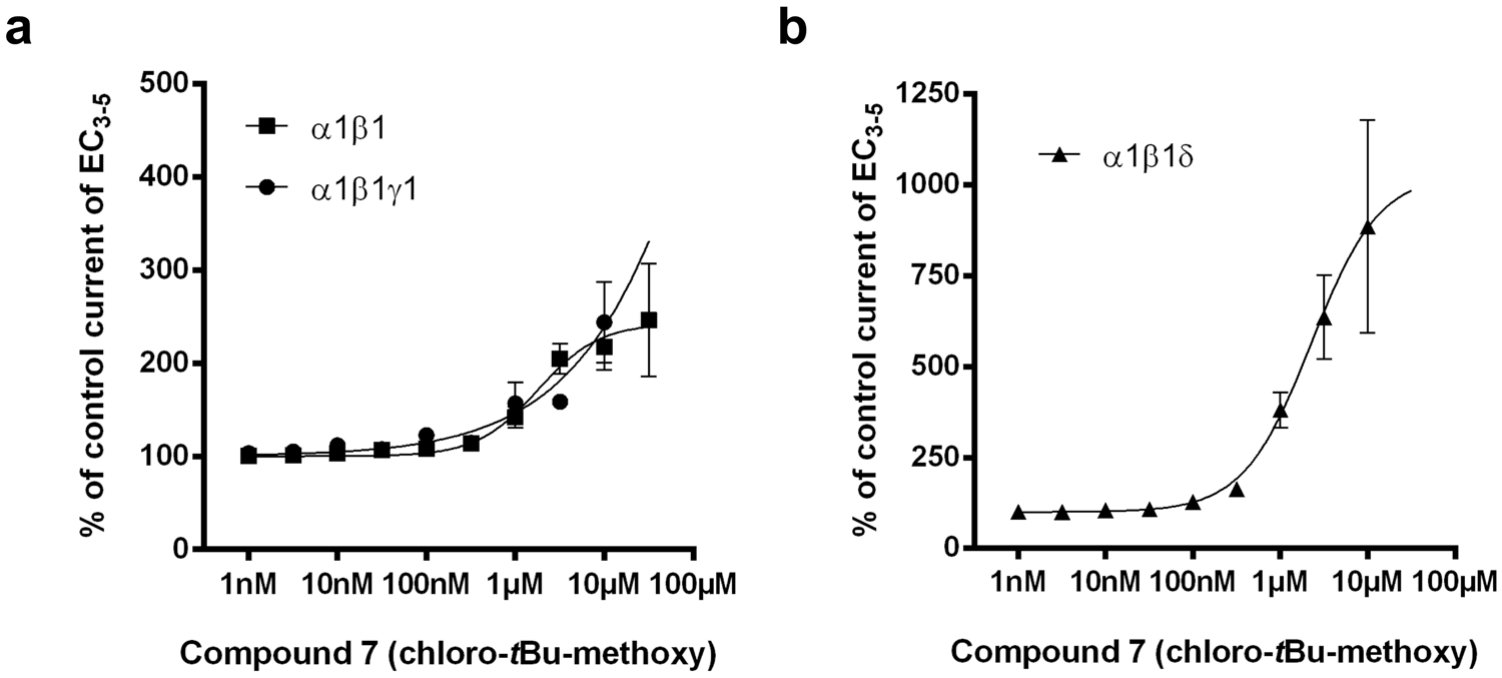
Compound **7** modulates GABA-evoked currents in α1β1, α1β1γ1 and α1β1δ receptors. (**a,b**) Concentration-dependent modulation of GABA EC_3–5_ current at α1β1, α1β1γ1 and α1β1δ receptors. Data represent means ± SEM (n = 4–17) (see Supplementary Table S19).

The resulting compound **7** (chloro-*t*Bu-methoxy; LAU462, see Fig. 1) has indeed no affinity for the benzodiazepine binding site (see Table 2), and was thus also tested functionally in α1β1γ1 and α1β1δ receptors. Figure 6 shows that it exerts modulatory effects quite similar to those of the parent compound **1**, but with an approximately twenty-fold right shift (see Supplementary Table S19 for data tables and Supplementary Fig. S20 for a sample trace). Again, we find no impact of the third subunit (γ1 or δ) on apparent potency.

In contrast to compound **1**, it modulates the α1β1δ receptor with higher efficacy (compare Fig. 5 to 6b). Accordingly, the R^6^ substituent that nearly abolished the sub-nanomolar affinity for the benzodiazepine site has a comparatively weaker impact on the potency at the α1+/β1− site. Thus, compound **7** serves as proof of principle for a potential development of ligands that target this binding site exclusively, and with useful potency.

## Discussion

Ligands that bind at α+/β− interfaces can in principle interact with amino acids unique to any α or any β isoform, and thus could theoretically be selective for any given combination of the 18 possible interfaces. We have previously reported strong functional α6 selectivity of some pyrazoloquinolinone ligands^22^. Here, we present pronounced potency preference for β1- containing receptors displayed by six R^8^ and R′^4^ substituted ligands tested in this study. Based on the most potent ligand **1** we synthesized an analogue with an additional R^6^ substituent that lacks the off-target interaction with the high affinity benzodiazepine site that is otherwise characteristic for many R^8^, R′^4^ substituted pyrazoloquinolinones.

Compound **1** is so far the most potent pyrazoloquinolinone ligand at any α+/β− interface, and represents an important lead towards the development of high affinity ligands with β1 specificity, while being largely unselective with respect to the principal α subunit. Compounds **3** and **4** also displayed high potency (~200 nM EC_50_) for the α1+/β1− interface.

The extracellular β1-, β2- and β3- minus sides all possess different amino acid sidechains that may contribute to the PQ binding site. In principle, this should provide the structural basis for potency differences to occur also between β2 and β3. In addition to the pronounced β1-preference, we also see a trend towards potency differences between β2- and β3-containing receptors. This raises hope that compounds can be identified with better potency and a wider window of separation between β2 and β3. Future libraries will aim to provide more insight into the ligand features that drive potency differences with respect to β isoforms.

It is interesting to compare the potency rank orders of the compounds between the different receptor subtypes: In previous studies^23^ we found that polar substituents in position R′^4^ enhance potency for the α1+/β3− interface, as reflected by the rank order **3** > **1** > **2**. Here we note that this rank order is also seen in the α1β2 receptor (see Fig. 3 and Supplementary Tables S1–S6). In contrast, the potency rank order for the α1β1 receptor is **1** > **3** > **2**, and, similarly for the R^8^ = methoxy series **4** > **6** > **5**. Thus, the requirements for high potency are different for β1 compared to β2 or β3. While the hydrophobic methyl group in R′^4^ is detrimental to potent interactions in all cases, the degree of polarity seems more important for the β2 and β3 isoforms, whereas the methoxy group performs best for the β1 isoform. It is interesting to note here that the methoxy group in **1** and **4** is an H-bond acceptor, while the amino group in **3** and **6** is an H-bond donor. It has to be investigated in future studies if this feature underlies the different potency rank orders in β1 versus β2,3.

These are not the first β1-selective ligands that are allosteric modulators of several GABA_A_ receptor subtypes. However, in comparison with the previously published β1 selective fragrant dioxane derivatives (FDDs)^16^, the PQs have the advantage of their known binding site, and higher potency (130 nM EC_50_ of compound **1** compared with 2500 nM of the most potent FDD). The previously described β1-selective partial negative modulator SCS on the other hand has very high potency^17^. While this compound has successfully been used in a number of interesting functional and biological assays, it so far has not been developed into a tool compound for the selective detection of β1-containing receptors. Moreover, its binding site in the TM domain is not known exactly, and it is also not yet known if it features a combined selectivity profile for certain other subunits, as it has been investigated only in a total of four receptor subtypes. We have tested the selective effects of the pyrazoloquinolinones presented here in a wider panel of receptor subtypes compared to FDDs or SCS.

The potential usefulness of highly potent PQ ligands with β1-selectivity is large. Along these lines, future efforts will be directed towards a detailed understanding of ligand features that reduce affinity for α+/γ2− while retaining and improving affinity for α+/β1−. The long term goal is to develop ligands which can be isotope labeled and used for the specific detection and quantification of β1-containing receptors. Here, the additional activity at the benzodiazepine binding site can be overcome readily for studies in *ex vivo* samples by blocking this site with any unlabeled high affinity benzodiazepine site ligand^35^. The described compounds offer good opportunities for isotopic labeling in the future. Compounds **1** and **4**–**7** contain a methoxy group, which can be used to introduce [^11^CH_3_] in the last stage of the synthesis, starting from the corresponding phenols. Furthermore, Schnürch and coworkers have published a proof of principle study for the tritiation of nitrogen containing heterocycles^36^, and this method can be applied to all described compounds. Radioligands will accelerate the testing of candidate compounds for α+/β− binding sites considerably, as the screening for new hits using functional assays is very slow.

Future applications of (suitably labelled) α+/β1− specific ligands are broad. For example, it has been discussed controversially whether cerebellar Purkinje cells express β1 subunits; Sergeeva and colleagues found no evidence, while Kelley *et al*. present evidence in favor^16, 37^. Tool compounds for the specific detection of α+/β1− interfaces in radioligand assays or autoradiographic studies, or with which receptors that contain this interface can be manipulated selectively in acute slices or in cultured neurons, could be helpful to investigate further. The pyrazoloquniolinone scaffold is also particularly attractive for the development of tool compounds to be used *in vivo*, such as experimental drugs for behavioral studies, or as PET ligands, because it already has been demonstrated to possess very low toxicity and adequate bio-availability^38^.

## Materials and Methods

### GABA_A_ receptor subunits and mutated subunits

cDNA’s of rat GABA_A_ receptor subunits α1, α4, β1, β2, β3 and γ2S were cloned as described^39^. cDNAs of the rat subunits α2, α3 and α5 were gifts from P. Malherbe, that of α6 and γ1 were gifts of P. Seeburg and that of δ was a gift of C. Czajkowski. The mutants were constructed using the Q5 Site-Directed Mutagenesis Kit (New England Biolabs) following manufacturer’s instruction. We used the wild-type rat β3-pCI vector as template and the primers GTGGGGATGAGGATCGACATCG and GCAGACTGGGGGACCCCC resulting in a substitution of amino acid N41 (AAC) to R (AGG). We used the wild-type rat β1-pCI vector as template and the primers CGTCGGGATGAACATCGATGTCGCC and TCCACCGGGGGCCCTCCA resulting in a substitution of amino acid R41 (CGG) to N (AAC). The mutated subunits were confirmed by sequencing.

### RNA Preparation

*In vitro* transcription of mRNA was based on the cDNA expression vectors encoding for rat GABA_A_ receptor subunits α1–6, β1-3, γ1,2, δ and the two β mutants (β1R41N and β3N41R)^40^. After linearizing the cDNA vectors with appropriate restriction endonucleases, the cDNA was purified and concentrated with the *DNA Clean and Contentrator* ^*TM*^ Kit (Zymoresearch, Catalog No. D4005). Capped transcripts of the purified cDNA were produced using the *mMESSAGE mMACHINE® T7* transcription kit (Ambion, TX, USA) and polyadenylated using the Ambion PolyA tailing kit (Ambion). After transcription and polyadenylation the RNA was purified with the *MEGAclear* ^*TM*^ Kit (Ambion, Catalog No. AM1908). The final RNA concentration was measured on *NanoDrop® ND-1000* and finally diluted and stored in diethylpyrocarbonate-treated water at −80 °C. For the microinjection, the RNA of αβ receptor combinations was mixed at 1:1 ratio (which leads to αβ receptors that consist of predominantly 3 beta and 2 alpha subunits^27^), for αkβlγm (k = 1–3, l = 1, 3, m = 1, 2) receptors at 1:1:5 ratio, for αkβ3γ2 (k = 4–6) and αkβlδ (k = 1, 4 and 6, l = 1, 3) receptor combinations at 3:1:5 ratio. All receptor combinations had a final concentration of 56 ng/µl.

### Two electrode voltage clamp (TEV) in *Xenopus laevis* oocytes

Mature female *Xenopus laevis* (Nasco, WI) were anesthetized in a bath of ice-cold 0.17% Tricain (Ethyl-m-aminobenzoate, Sigma, MO) before decapitation in full accordance with all rules of the Austrian animal protection law (see http://www.ris.bka.gv.at/Dokumente/BgblAuth/BGBLA_2012_I_114/BGBLA_2012_I_114.pdf) and the Austrian animal experiment by-laws (see https://www.ris.bka.gv.at/Dokumente/BgblAuth/BGBLA_2012_II_522/BGBLA_2012_II_522.pdf) which implement the European Directive 2010/63/EU (see http://eur-lex.europa.eu/LexUriServ/LexUriServ.do?uri=OJ:L:2010:276:0033:0079:en:PDF) into the Austrian law (all information accessed on July 27, 2016). The frog’s ovaries were transferred to ND96 medium (96 mM NaCl, 2 mM KCl, 1 mM MgCl2, 5 mM HEPES; pH 7.5). Stage 5–6 oocytes with the follicle cell layer around them were roughly dissected with forceps into packages of 10–15 cells and washed in Ca^2+^-free ND96 medium. Cells were then digested with collagenase (type IA, Sigma, NO, 1 mg/mL ND96) at 18 °C shaking at 30 rpm for 30–60 minutes and gently defolliculated with the aid of a glass pipette with appropriate tip diameter and a platinum loop. Defolliculated cells were stored at 18 °C for at least 6 hours in ND96 solution containing penicillin G (10000 IU/100 mL) and streptomycin (10 mg/100 mL) in order to preselect and exclude damaged cells from further treatment. Healthy defolliculated oocytes were injected with an aqueous solution of mRNA. A total of 4.5 ng of mRNA per oocyte was injected with a Nanoject II (Drummond). After injection of mRNA, oocytes were incubated at 18 °C (ND96 + antibiotic) for 2–3 days for αβ receptors and for 3–4 days for αβγ or αβδ receptors before recording. When cells were measured at later time points, oocytes were stored at +4 °C instead of 18 °C.

For electrophysiological recordings, oocytes were placed on a nylon-grid in a bath of Ca^2+^-containing NDE solution medium [96 mM NaCl, 5 mM HEPES-NaOH (pH 7.5), 2 mM KCl, 1 mM MgCl_2_, 1.8 mM CaCl_2_]. For current measurements the oocytes were impaled with two microelectrodes (1–3 MΩ resistance) filled with 2 M KCl. The oocytes were constantly washed by a flow of 6 mL/min NDE that could be switched to NDE containing GABA and/or drugs. The EC_3–5_ was determined at the beginning of each experiment. Drugs were diluted into NDE from DMSO-solutions resulting in a final concentration of 0.1% DMSO perfusing the oocytes. Compounds were co-applied with GABA until a peak response was observed. Between two applications, oocytes were washed in NDE for up to 15 min to ensure full recovery from desensitization. Maximum currents measured in mRNA injected oocytes were in the microampere range for all subtypes of GABA_A_ receptors. To test for modulation of GABA induced currents by drugs a concentration of GABA that was titrated to trigger 3–5% of the respective maximum GABA-elicited current of the individual oocyte (EC_3–5_) was applied to the cell with increasing concentrations of compounds. In order to monitor receptor composition, diazepam (~200% modulation at 1 µM) was used to investigate the incorporation of the γ2^25^ subunit, DS2 (>800% modulation at 1 µM) for the incorporation of the δ subunit and triazolam (>200% modulation at 10 µM) for the γ1 incorporation^41^. Enhancement of the chloride current was defined as (I_GABA+Comp_/I_GABA_) - 1, where I_GABA+Comp_ is the current response in the presence of a given compound and I_GABA_ is the control GABA current. All recordings were performed at room temperature at a holding potential of 60 mV using a Dagan TEV-200A two-electrode voltage clamp (Dagan Corporation, Mineapolis, MN). Data were digitized, recorded and measured using an Axon Digidata- 1550 low-noise data acquisition system (Axon Instruments, Union City, CA). Data acquisition was done using pCLAMP v.10.5 (Molecular Devices™, Sunnyvale, CA).

Data were analysed using GraphPad Prism v.6. and plotted as concentration-response curves. These curves were normalized and fitted by non-linear regression analysis to the equation Y = bottom + (top-bottom)/1+10^(LogEC50-X)*nH^, where EC_50_ is the concentration of the compound that increases the amplitude of the GABA-evoked current by 50%, and nH is the Hill coefficient. Data are given as mean ± SEM from at least three oocytes of two and more oocyte batches. Statistical significance was calculated using an extra sum of squares *F*-Test (see Figs 3 and 4). *P*-values of <0.05 were accepted as statistically significant.

### Radioligand displacement assays

Rat cerebellar membranes were prepared and radioligand binding assays were performed as described previously^42^. In brief, membrane pellets were incubated for 90 min at 4 °C in a total of 500 µL of a solution containing 50 mM Tris/citrate buffer, pH = 7.1, 150 mM NaCl and 2 nM [^3^H]flunitrazepam in the absence or presence of either 5 µM diazepam (to determine unspecific binding) or various concentrations of receptor ligands (dissolved in DMSO, final DMSO-concentration 0.5%). Membranes were filtered through Whatman GF/B filters and washed twice with 4 mL of ice-cold 50 mM Tris/citrate buffer. Filters were transferred to scintillation vials and subjected to scintillation counting after the addition of 3 mL Rotiszint Eco plus liquid scintillation cocktail. Nonlinear regression analysis of the displacement curves used the equation: log(inhibitor) vs. response - variable slope with Top = 100% and Bottom = 0% Y = 100/(1 + 10^((logIC_50_-x)*Hillslope)).

Saturation binding experiments were performed by incubating the membranes with various concentrations of [^3^H]flunitrazepam in the absence or presence of 5 µM diazepam and analyzed using the equation Y = Bmax*X/(KD + X) and an equilibrium binding constant KD for rat cerebellum was determined (SD ± SEM n = 3 independent experiments): 4.8 ± 0.3 nM

IC_50_ values were converted to Ki values using the Cheng-Prusoff relationship^43^ Ki = IC_50_/(1 + (S/KD)) with S being the concentration of the radioligand (2 nM) and the KD value described above (4.8 nM).

All analyses were performed using GraphPad Prism version 7 for PC, GraphPad Software, La Jolla California USA, www.graphpad.com.

### Investigated compounds

We tested pyrazoloquinolinones with combined substituents at the position R^8^ and R^6^ on ring A (Cl, OMe and *t*Bu) and R′^4^ position on ring D (methoxy, methyl, amino). The following compounds were used: Compound **1** (PZ II 028): C_17_H_12_ClN_3_O_2_: 8-Chloro-2-(4-methoxyphenyl)-2H-pyrazolo[4,3-c]quinolin-3(5H)-one [=Cpd 11]^23^, Compound **2** (LAU156): C_17_H_12_ClN_3_O: 8-Chloro-2-(4-methylphenyl)-2H-pyrazolo[4,3-c]quinolin-3(5H)-one [=Cpd 10]^23^; Compound **3** (LAU206): C_16_H_11_ClN_4_O: 8-Chloro-2-(4-aminophenyl)-2H-pyrazolo[4,3-c]quinolin-3(5H)-one [=Cpd 13]^23^; Compound **4** (LAU176): C_18_H_15_N_3_O_3_ **:** 8-Methoxy-2-(4-methoxyphenyl)-2,5-dihydro-3H-pyrazolo[4,3-c]quinolin-3-one; Compound **5** (DCBS76): C_18_H_15_N_3_O_2_: 8-Methoxy-2-(4-methylphenyl)-2,5-dihydro-3H-pyrazolo[4,3-c]quinolin-3-one; Compound **6** (DCBS96): C_17_H_14_N_4_O_2_: 2-(4-Aminophenyl)-8-methoxy-2,5-dihydro-3H-pyrazolo[4,3-c]quinolin-3-one; Compound **7** (LAU462): C_21_H_20_ClN_3_O_2_: 6-(tert-Butyl)-8-chloro-2-(4-methoxyphenyl)-2,5-dihydro-3H-pyrazolo[4,3-c]quinolin-3-one; Compound **8** (Diazepam) (Sigma-Aldrich, St. Louis, MO, USA); Compound **9** (Triazolam) (Sigma-Aldrich, MO, USA); Compound **10** (DS2) (R&D Systems, MN, USA).

### Compound synthesis

Commercially available reagents were used without further purification. Reactions were monitored by thin layer chromatography with silica gel 60 F_254_ plates (E. Merck, Darmstadt, Germany). HPLC chromatography was carried out with the Autopurification system by Waters using fluoro-phenyl columns. ^1^H and ^13^C NMR spectra were recorded on Bruker *AC 200* (^1^H: 200 MHz, ^13^C: 50 MHz), Bruker *Avance Ultrashield 400* (^1^H: 400 MHz, ^13^C: 101 MHz) or Bruker *Avance IIIHD 600* spectrometer equipped with a Prodigy BBO cryo probe (^1^H: 600 MHz, ^13^C: 151 MHz). Chemical shifts are reported in parts per million (ppm) and were calibrated using DMSO-*d*_6_ as internal standard. Multiplicities are denoted by s (singlet), br s (broad singlet), d (doublet), dd (doublet of doublet) and m (multiplet). Melting points were determined with a Büchi Melting Point B-545 apparatus. HR-MS was measured on an Aglient 6230 LC TOFMS mass spectrometer equipped with an Aglient Dual AJS ESI-Source.

Compounds **1** (PZ II 028), **2** (LAU156), **3** (LAU206) and **4** (LAU176) were synthesized and published previously^23^. Synthesis of **5** (DCBS76) was conducted in analogy to previously outlined synthetic routes^23, 44, 45^. The synthesis of **6** (DCBS96) was improved as described. Compound **7** (LAU462) was synthesized according to reported protocols^23, 46, 47^.

#### 8-Methoxy-2-(4-methylphenyl)-2,5-dihydro-3H-pyrazolo[4,3-c]quinolin-3-one **5** (DCBS76)

Compound **5** was synthesized according to the literature^23, 44, 45^ in 74% yield (yellow solid, 85 mg, 0.28 mmol). ^1^H NMR (400 MHz, DMSO-*d*_6_) δ 2.32 (s, 3H), 3.93 (s, 3H), 7.22–7.27 (m, 2H), 7.29 (dd, *J* = 9.1, 2.9 Hz, 1H), 7.58 (d, *J* = 2.8 Hz, 1H), 7.67 (d, *J* = 9.0 Hz, 1H), 8.08–8.16 (m, 2H), 8.65 (s, 1H), 12.79 (br s, 1H). ^13^C NMR (101 MHz, DMSO-*d*_6_) δ 20.5, 55.7, 102.5, 105.3, 118.7 (2 C), 119.6, 120.0, 121.2, 129.0 (2 C), 129.7, 132.9, 137.8, 137.9, 142.7, 157.5, 161.4. HR-MS: calculated [C_18_H_16_N_3_O_2_^+^]: 306.1237; found [C_18_H_16_N_3_O_2_^+^]: 306.1230 (diff.: 2.31 ppm). TLC (10% MeOH in CH_2_Cl_2_): R_f_ = 0.54. M.p.: decomposes > 300 °C.

#### 2-(4-Aminophenyl)-8-methoxy-2,5-dihydro-3H-pyrazolo[4,3-c]quinolin-3-one **6** (DCBS96)

8-Methoxy-2-(4-nitrophenyl)-1,2-dihydro-3H-pyrazolo[4,3-c]quinolin-3-one (20 mg, 0.06 mmol) was dissolved in 2.5 mL MeOH, Pd/C (10 wt-%) was added and the reaction mixture was stirred at room temperature under hydrogen atmosphere. After 18 h the reaction mixture was passed through a bed of silica and the solvent was removed under reduced pressure. The residue was purified by HPLC and neutralized with 1 mL satd. NaHCO_3_. The precipitate was washed with water (2 × 2 mL) and dried in *vacuo* to give 2-(4-aminophenyl)-8-methoxy-2,5-dihydro-3H-pyrazolo[4,3-c]quinolin-3-one as yellow solid (16 mg, 0.052 mmol, 87%). ^1^H NMR (600 MHz, DMSO-*d*_6_) δ 3.90 (s, 3H), 4.99 (br s, 2H), 6.58–6.64 (m, 2H), 7.21 (dd, *J* = 9.0, 2.9 Hz, 1 H), 7.52 (d, *J* = 2.9 Hz, 1H), 7.63 (d, *J* = 9.0 Hz, 1H), 7.78–7.83 (m, 2H), 8.54 (s, 1H). ^13^C NMR (151 MHz, DMSO-*d*_6_) δ 55.6, 102.3, 105.4, 113.6 (2C), 119.2, 120.2, 120.9 (2C), 121.5, 129.9, 130.0, 137.6, 142.0, 145.6, 157.3, 160.6. HR-MS: calculated [C_17_H_15_N_4_O_2_^+^]: 307.1190; found [C_17_H_15_N_4_O_2_^+^]: 307.1196 (diff.: −2.21 ppm). TLC (5% MeOH in CH_2_Cl_2_): R_f_ = 0.25. M.p.: decomposes > 300 °C.

#### 6-(*tert*-Butyl)-8-chloro-2-(4-methoxyphenyl)-2*H*-pyrazolo[4,3-*c*]quinolin-3(5*H*)-one **7** (LAU462)

Compound **7** was synthesized according to the literature^44, 45, 48^ in 24% yield (yellow solid, 83 mg, 0.22 mmol). ^1^H NMR (400 MHz, DMSO-*d*_6_) δ 1.54 (s, 9H), 3.79 (s, 3H), 7.02 (d, *J* = 9.1 Hz, 2H), 7.55 (d, *J* = 2.2 Hz, 1H), 8.09 (m, 3H), 8.41 (d, *J* = 6.8 Hz, 1H), 11.08 (d, *J* = 6.8 Hz). ^13^C NMR (101 MHz, DMSO-*d*_6_) δ 30.3, 35.1, 55.7, 106.5, 114.3, 119.9, 120.8, 121.5, 128.0, 130.9, 131.9, 133.8, 138.8, 142.1, 142.6, 156.5, 161.1. HR-MS: calculated [C_21_H_21_ClN_3_O_2_^+^]: 382.1323; found [C_21_H_21_ClN_3_O_2_^+^]: 382.1334 (diff.: 4.50 ppm). TLC (10% EtOAc in CH_2_Cl_2_): R_f_ = 0.67. M.p.: decomposes > 298 °C.

### Data Availability

The datasets generated during and/or analysed during the current study are available from the corresponding author upon request.

## Electronic supplementary material

Supplementary Information
