# Supplementary material for: Molecular tools for GABAA receptors: High affinity ligands for β1-containing subtypes
Source: Sci Rep. 2017 Jul 18;7:5674. doi: 10.1038/s41598-017-05757-4 (PMC5516028; doi:10.1038/s41598-017-05757-4)
Supplement: Supplementary file 1 — Supplementary Information [file 41598_2017_5757_MOESM1_ESM.pdf]

Supplementary Information

Molecular tools for $GABA_A$ receptors: High affinity ligands for β1-containing subtypes

Xenia Simeone (1), David C. B. Siebert (2), Konstantina Bampali (1), Zdravko Varagic (1), Marco Treven (1), Sabah Rehman (1), Jakob Pyszkowski (1), Raphael Holzinger (1), Friederike Steudle (1), Petra Scholze (3), Marko D. Mihovilovic (2), Michael Schnürch (2), Margot Ernst* (1)

(1) Department of Molecular Neurosciences, Center for Brain Research, Medical University Vienna, Spitalgasse 4, 1090 Vienna, Austria.

(2) Institute of Applied Synthetic Chemistry, TU Wien, Getreidemarkt 9/163, 1060 Vienna, Austria

(3) Department of Pathobiology of the Nervous System, Center for Brain Research, Medical University of Vienna, Spitalgasse 4, 1090 Vienna, Austria

Contact: margot.ernst@meduniwien.ac.at

Supplementary Table S1:

PZ II 028 (**1**)	α1β1	α1β2	α1β3
EC_{50} [µM]	0.13	1.15	1.84
$LogEC_{50}$	-6.89±0.1	-5.94±0.1	-5.7±0.1
nH	1.3±0.4	1.1±0.3	1.5±0.5
1 nM	101.7±1.7	100.0±0	101.1±1.5
3 nM	100.5±0.5	-	-
10 nM	122.3±5.2	129.3±4.5	105.5±1.6
30 nM	137.5±13.3	172.8±5.1	-
100 nM	243.6±24.7	290.8±9.5	136.0±4.9
300 nM	291.5±24.5	501.0±33.6	209.8±11.6
1 µM	418.5±20.2	817.0±55.4	392.4±33.8
3 µM	416.3±39.9	926.0±84.2	839.2±104.8
10 µM	377.0±29.4	643.5±57.0	1063.0±89.9
30 µM	174±34.2	-	900.4±88.4
n	4-7	4	3-8

EC_{50}, Hill slope (nH) and efficacy of compound **1** (PZ II 028) at increasing concentrations in α1β1, α1β2 and α1β3 receptors. The dose-response curves of Figure 3 were prepared from this data, which is reported as mean ± SEM. Control current = 100% (GABA $EC_{3\text{-}5}$).

Supplementary Table S2:

LAU156 (**2**)	α1β1	α1β2	α1β3
EC_{50} [µM]	0.77	4.31	2.39
$LogEC_{50}$	-6.12±0.2	-5.37±0.1	-5.62±0.1
nH	1.0±0.4	2.0±0.9	1.9±0.6
1 nM	-	-	100.1±0.1
3 nM	94.5±2.2	-	-
10 nM	99.6±3.7	-	100.7±0.5
30 nM	104.4±5.1	102.1±1.0	-
100 nM	128.7±10.8	104.9±1.4	109.0±2.2
300 nM	194.5±23.3	109.7±3.3	122.5±5.4
1 µM	277.2±37.7	133.6±8.2	151.2±11.5
3 µM	365.3±44.8	194.1±16.4	276.2±41.7
10 µM	405.3±49.8	335.2±48.0	372.3±40.1
30 µM	414.8±50.7	362.6±46.9	374.9±34.7
n	6-8	6	6-8

EC_{50}, Hill slope (nH) and efficacy of compound **2** (LAU156) at increasing concentrations in α1β1, α1β2 and α1β3 receptors. The dose-response curves of Figure 3 were prepared from this data, which is reported as mean ± SEM. Control current = 100% (GABA $EC_{3\text{-}5}$).

Supplementary Table S3:

LAU206 (**3**)	α1β1	α1β2	α1β3
EC_{50} [µM]	0.22	1.05	1.47
$LogEC_{50}$	-6.66±0.2	-5.98±0.1	-5.83±0.1
nH	1.0±0.4	2.0±0.7	1.1±0.5
1 nM	-	-	106.1±1.9
3 nM	115.9±5.9	-	-
10 nM	115.6±3.6	-	107.5±5.1
30 nM	141.0±11.2	104.5±4.0	-
100 nM	210.0±16.8	115.2±6.2	125.3±4.6
300 nM	298.6±29.7	133.9±3.7	136.3±8.5
1 µM	353.0±41.8	169.8±5.3	176.4±18.7
3 µM	433.4±47.0	243.8±8.3	292.9±19.5
10 µM	402.7±50.9	238.0±16.1	318.4±18.4
30 µM	343.9±32.5	105.8±11.4	163.3±2.3
n	3-4	4	3-5

EC_{50}, Hill slope (nH) and efficacy of compound **3** (LAU206) at increasing concentrations in α1β1, α1β2 and α1β3 receptors. The dose-response curves of Figure 3 were prepared from this data, which is reported as mean ± SEM. Control current = 100% (GABA EC_{3-5}).

Supplementary Table S4:

LAU176 (**4**)	α1β1	α1β2	α1β3
EC_{50} [µM]	0.21	2.62	3.13
$LogEC_{50}$	-6.68±0.1	-5.58±0.1	-5.51±0.1
nH	1.8±0.4	1.3±0.5	1.9±0.5
1 nM	100.0±0	56.2±0.2	99.0±4.2
3 nM	100.0±0	100.0±0	-
10 nM	111.3±2.4	82.4±23.2	108.4±4.5
30 nM	127.0±1	100.0±0	-
100 nM	183.0±19.9	110.4±13.8	119.6±5.7
300 nM	396.8±54.9	165.6±7.9	148.7±15.8
1 µM	470.2±14.1	331.4±37.4	242.0±16.0
3 µM	546.7±14.6	689.3±82.9	653.7±109.1
10 µM	507.5±21.3	1050.4±107.4	1124.8±103.8
30 µM	218.0±11.0	1125.5±194.5	1139.4±74.7
n	3-9	3-7	3-8

EC_{50}, Hill slope (nH) and efficacy of compound **4** (LAU176) at increasing concentrations in α1β1, α1β2 and α1β3 receptors. The dose-response curves of Figure 3 were prepared from this data, which is reported as mean ± SEM. Control current = 100% (GABA EC_{3-5}).

Supplementary Table S5:

DCBS76 (**5**)	α1β1	α1β2	α1β3
EC_{50} [µM]	0.71	3.74	3.49
$LogEC_{50}$	-6.15±0.2	-5.43±0.2	-5.46±0.2
nH	1.0±0.5	2.8±1.7	2.7±1.4
1 nM	-	-	-
3 nM	109.8±3.2	100.0±0	105.0±3.1
10 nM	123.0±0	108.3±2.5	111.4±5.7
30 nM	145.3±5.3	111.0±4.2	115.4±8.1
100 nM	192.3±5.7	113.6±10.6	126.5±11.5
300 nM	313.9±16.9	136.9±5.6	133.9±12.4
1 µM	429.5±52.9	178.9±8.3	183.3±24.4
3 µM	602.7±90.7	363.0±18.3	383.7±47.4
10 µM	731.5±141.2	817.9±87.8	719.9±65.5
30 µM	637.1±114.3	651.3±147.7	623.4±113.6
n	3-5	3-4	5-6

EC_{50}, Hill slope (nH) and efficacy of compound **5** (DCBS76) at increasing concentrations in α1β1, α1β2 and α1β3 receptors. The dose response curves of Figure 3 were prepared from this data, which is reported as mean ± SEM. Control current = 100% (GABA EC_{3-5}).

Supplementary Table S6:

DCBS96 (**6**)	α1β1	α1β2	α1β3
EC_{50} [µM]	0.65	n.d.	n.d.
$LogEC_{50}$	-6.19±0.1	n.d.	n.d.
nH	1.3±0.2	n.d.	n.d.
1 nM	-	-	-
3 nM	100.8±1.5	95.7±2.3	100.5±1.9
10 nM	99.8±1.7	99.7±2.0	100.8±0.8
30 nM	100.0±0	98.0±3.0	99.3±2.7
100 nM	115.5±5.0	99.5±5.5	95.0±5.0
300 nM	153.2±9.5	97.3±6.4	98.8±2.8
1 µM	212.1±9.5	97.5±9.5	97.5±0.5
3 µM	263.2±9.1	107.7±4.5	103.8±1.1
10 µM	265.9±14.5	118.0±9.0	115.8±7.2
30 µM	214.8±7.8	105.3±8.3	107.2±1.0
n	4	3	3-6

EC_{50}, Hill slope (nH) and efficacy of compound **6** (DCBS96) at increasing concentrations in α1β1, α1β2 and α1β3 receptors. Dose-response curves could not be fitted for α1β2 and α1β3 due to lack of efficacy. Note that the compound changes trace shape, and thus is interacting with these receptors – but not as a positive modulator. The dose-response curve for α1β1 of Figure 3 was prepared from this data, which is reported as mean ± SEM. Control current = 100% (GABA EC_{3-5}).

Supplementary Figure S7:

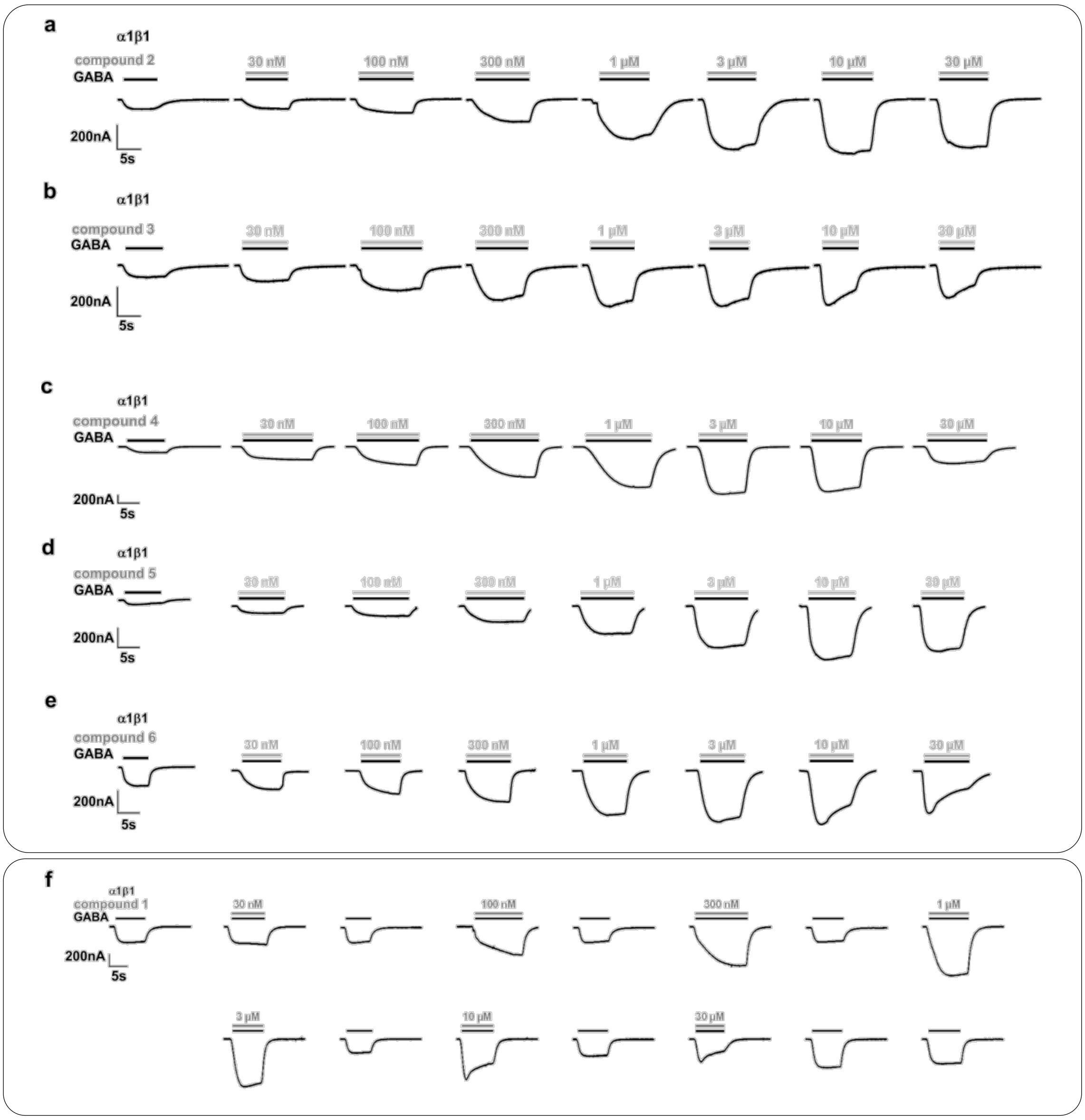

Original current traces showing the potentiation of the GABA-elicited response (EC_{3-5}) by increasing concentrations of compounds **2-6** (**a-e**) in α1β1 receptors. In **f** the effects of increasing concentrations of compound **1** are shown, with GABA EC_{3-5} control applied between each co-application of GABA EC_{3-5} and compound **1**. This trace shows that rise time of GABA currents is faster than for GABA-compound co-application.

Supplementary Figure S8:

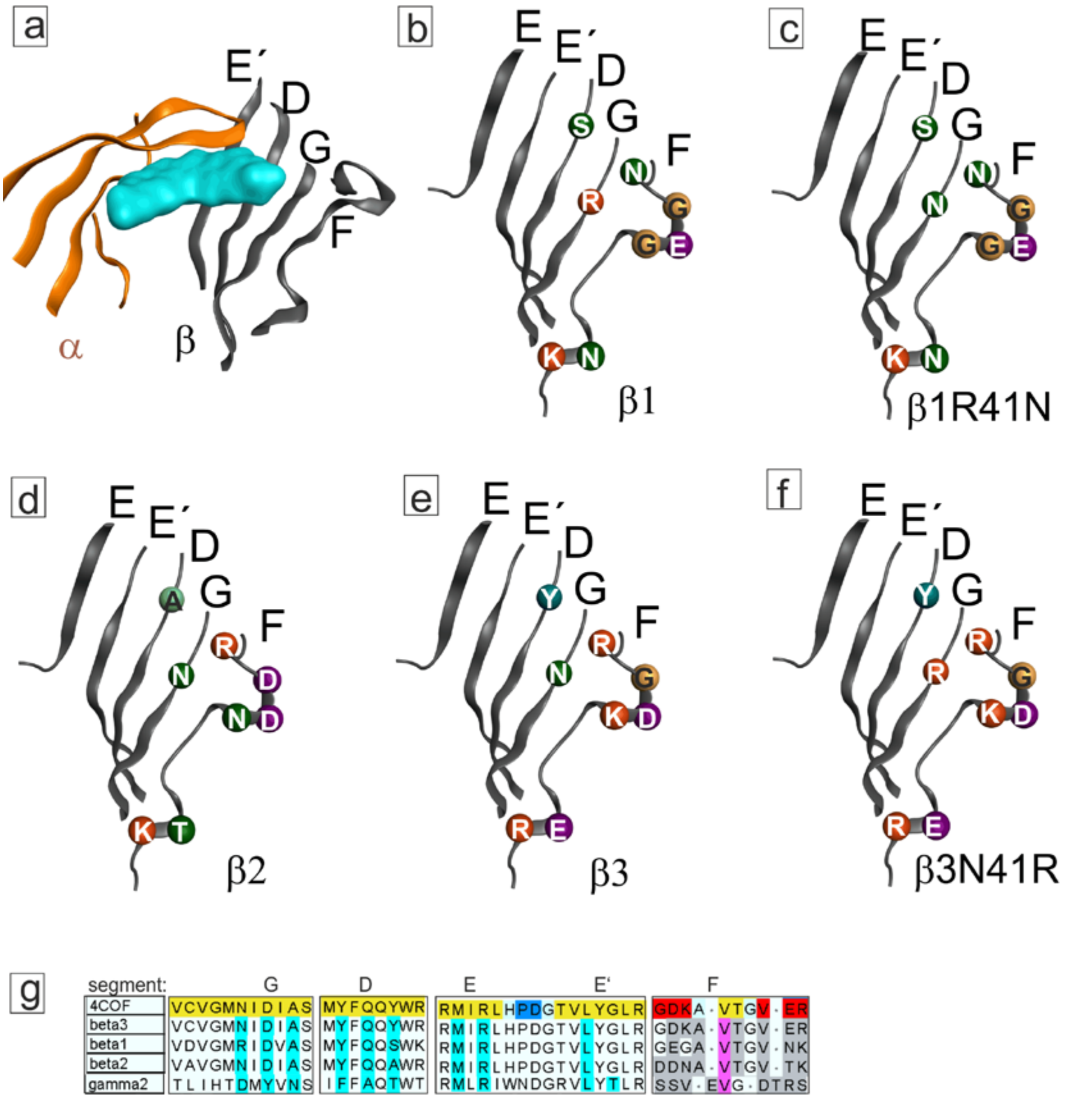

Homology models of the structural differences at the β- half pocket of the three β isoforms. a: View of the entire binding site at the extracellular α+ (light brown) β- (grey) with the predicted ligand occupied volume shown as cyan space filling surface. The amino acid at position 41 of loop G (β1, β3 counting of rat mature protein) is localized most central to the pocket among the variable amino acids. All other minus side amino acids are identical in all three isoforms. **b, d, e:** Minus sides of the three beta isoforms. Color coding of the amino acids is according to the ClustalX color scheme. **c, f**: Minus sides of the two mutants β1R41N and β3N41R. **g:** Alignment of segments (“loops”) G, D, E and F of the three β- isoforms with the γ2 sequence and with the 4COF sequence to indicate homologous positions. Color code for 4COF: beta strands: yellow; helices: red; turns: blue. Color code for the subunit sequences: cyan indicates pocket forming positions, magenta indicates a structurally conserved position in “loop F”, grey indicates structurally uncertain positions due to low homology.

Supplementary Figure S9: GABA DR curves

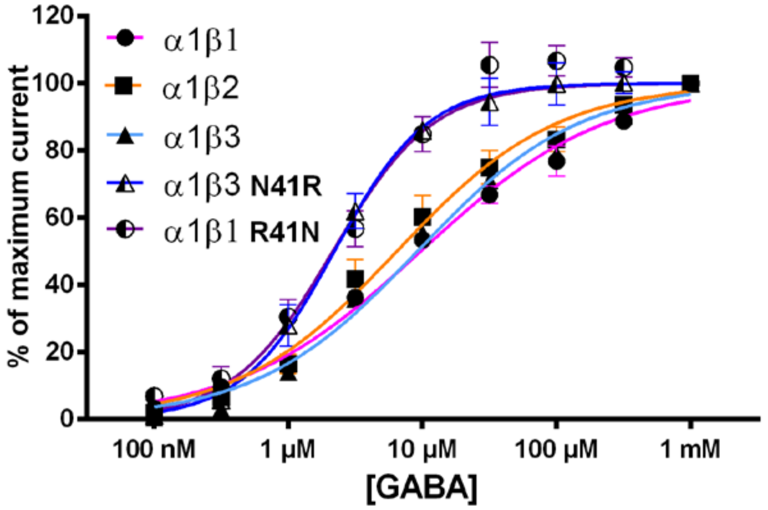

GABA dose-response curves in α1β1 (● filled dots, magenta line), α1β1R41N (half filled dots, purple line), α1β2 (■ filled squares, orange line), α1β3 (▲ filled triangles, blue line) and α1β3N41R (half filled triangles, dark blue line) $GABA_A$ receptors.

Supplementary Table S10: Data of GABA DR curves

GABA	α1β1	α1β2	α1β3	α1β3N41R	α1β1R41N
EC_{50} (µM)	9.7	6.4	9.2	2.2	2.2
$LogEC_{50}$	-5.01±0.03	-5.19±0.05	-5.04±0.02	-5.65±0.04	-5.67±0.05
nH	0.6±0.3	0.7±0.1	0.7±0.0	1.2±0.1	1.2±0.1
amplitude (µA)					
100 nM	0.1±0.0	0.0±0.0	0.0±0.0	0.0±0.0	0.1±0.0
1 µM	0.7±0.1	0.5±0.2	0.5±0.1	1.3±0.5	0.7±0.1
10 µM	2.2±0.3	1.9±0.5	1.9±0.3	3.5±0.9	2.3±0.5
100 µM	3.1±0.4	2.6±0.6	2.7±0.4	4.0±1.1	2.9±0.7
1 mM	4.0±0.5	3.1±0.7	3.5±0.5	4.1±1.2	2.8±0.8
n	9	5	10	4	6

EC_{50}, Hill slope (nH) and efficacy of GABA at increasing concentrations in α1β1, α1β2, α1β3 and the mutant receptors. Supplementary Fig. S9 was prepared from this data, which is reported as mean ± SEM.

Supplementary Table S11:

	Cpd 1	Cpd 2	Cpd 3	Cpd 4	Cpd 5	Cpd 6
EC_{50} (µM)	0.8	n.d.	n.d.	1.2	n.d.	n.d.
$LogEC_{50}$	-6.10±0.13	n.d.	n.d.	-5.9±0.23	n.d.	n.d.
% of control current at EC_{3-5}						
100nM	80±10	70±5	70±1	80±10	95±20	75±1
300nM	100±10	62±8	65±6	85±12	85±25	90±10
1µM	140±16	62±8	85±7	110±20	100±30	90±15
3µM	195±20	60±9	100±8	150±25	120±30	90±20
10µM	205±20	60±10	125±5	180±45	155±40	70±10
30µM	150±10	60±8	110±10	140±15	160±40	115±1
n	4	4	4	3	3	3

EC_{50} and efficacy of compounds **1-6** at increasing concentrations in α1β1R41N receptors. Data are reported as mean ± SEM. Control current = 100% (GABA EC_{3-5}).

Supplementary Figure S12:

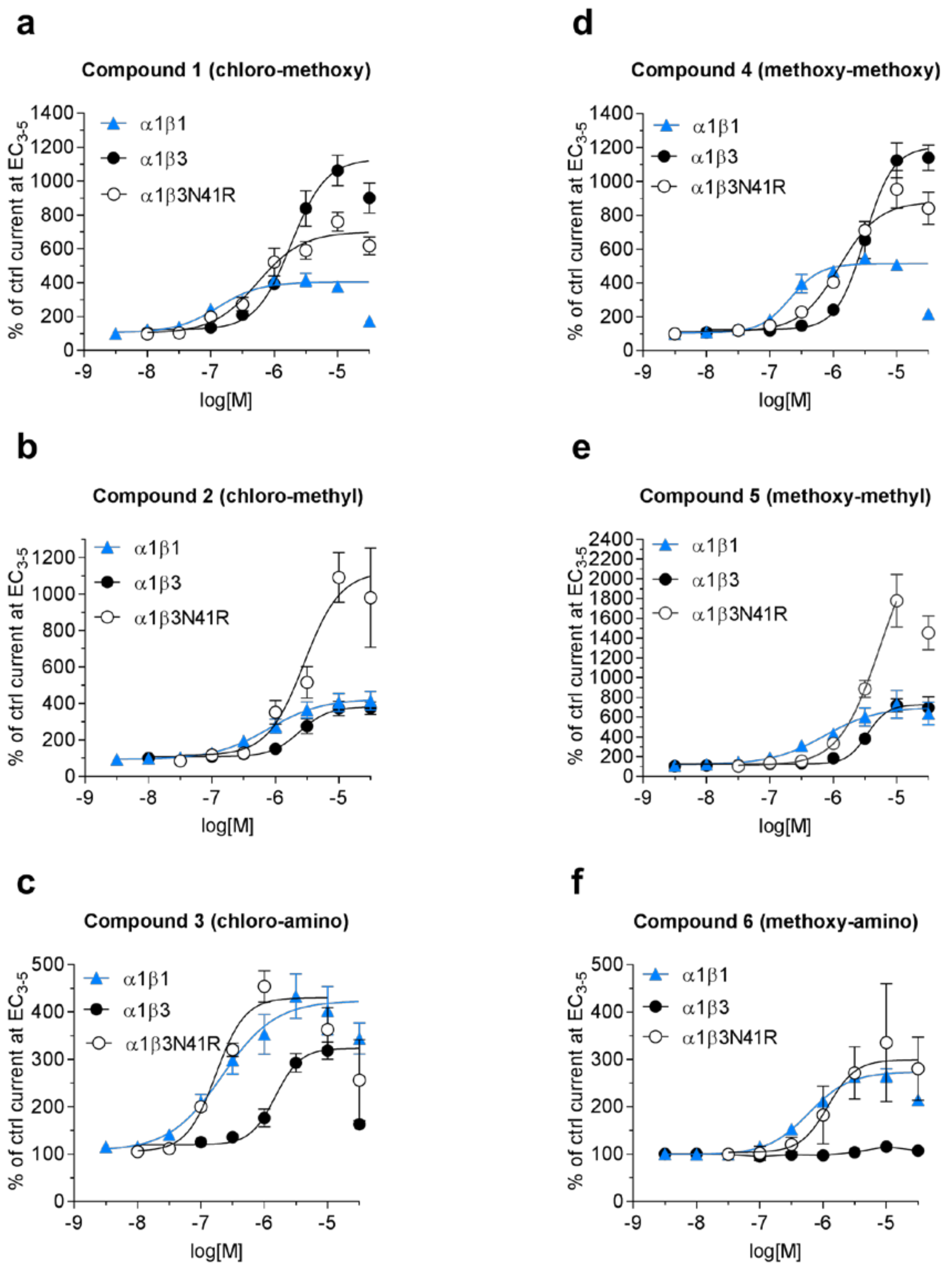

Dose-response curves of compounds **1-6** co-applied with GABA EC_{3-5} in α1β3R41N (**a-f**). Dose-response curves for α1β1 and α1β3 are also shown for comparison (reproduced from Figure 3). In two instances (**b, e**) where high compound concentrations elicited substantial desensitization, the highest compound concentration (30 µM) was excluded from the fit.

Supplementary Table S13:

	EC$_{50}$ (μM)					
	**α1βlγ2**	**α2βlγ2**	**α3βlγ2**	**α4βlγ2**	**α5βlγ2**	**α6βlγ2**
**β1**	0.1	0.05	n.d.	n.d.	0.05	-
**β2**	1.9	3.5	2.1	-	3.0	-
**β3**	1.8	2.2	1.5	0.9	0.6	3.3
	**α1β3**	**α2β3**	**α3β3**	**-**	**α5β3**	**-**
	1.6	3.0	1.8	-	0.6	-

Approximate EC$_{50}$ values (published data[1]) of compound **1** (PZ II 028) in αkβlγ2 (k= 1-6; l= 1-3) and in αkβ3 (k=1,2,3 and 5) receptors; n.d. not determined - efficacy was too low to perform the curve fit.

Supplementary Table S14:

LAU156 (**2**)	α1β3γ2	α2β3γ2	α3β3γ2	α4β3γ2	α5β3γ2	α6β3γ2
**EC$_{50}$[μM]**	**4.4**	**5.5**	**7.2**	**13.4**	**6.4**	**>5**
LogEC$_{50}$	5.4±0.3	5.3±0.2	5.1±0.7	4.9±0.6	5.2±0.4	n.d.
1 nM	100.0±0	100.0±0	100.0±0	100.9±1.8	93.3±5.7	105.6±6.6
3 nM	-	-	-	96.3±8.0	-	-
10 nM	108.0±4.0	108.1±4.2	106.7±6.7	100.4±2.2	95.1±6.9	114.5±8.8
30 nM	-	-	-	104.4±4.4	-	-
100 nM	108.0±4.0	111.1±2.3	109.0±1.0	119.8±10.1	97.7±11.5	152.1±12.0
300 nM	127.9±5.6	133.5±0		128.0±4.7	102.6±14.9	
1 μM	155.9±19.0	136.6±5.5	148.0±8.7	161.9±16.3	122.8±16.5	296.9±16.2
3 μM	314.0±83.3	-	-	178.0±4.7	-	-
10 μM	423.1±106.5	470.5±50.0	305.3±71.0	267.1±16.8	220.1±31.1	1482.9±158.3
30 μM	533.6±136.2	579.7±0	385.0±99.9	314.9±30.1	265.9±41.0	-
n	3	3-9	3	3	3	6-8

EC$_{50}$ (bold-faced) and efficacy of compound **2** (LAU156) at increasing concentrations in αkβ3γ2 (k=1-6) receptors. Compound **2** modulates all receptors but with low efficacy with EC$_{50}$ values ranging from 4.4 μM (α1β3γ2) to 13.4 μM (α4β3γ2). Saturation could not be reached in α6β3γ2 thus EC$_{50}$ values could not be obtained. Table 1 was prepared from this data. Control current = 100% (GABA EC$_{3-5}$; n.d.=not determined).

Supplementary Table S15:

LAU206 (**3**)	α1β3γ2	α2β3γ2	α3β3γ2	α4β3γ2	α5β3γ2	α6β3γ2
**EC_{50} [µM]**	**1.0**	**n.d.**	**1.2**	**n.d.**	**n.d.**	**2.3**
$LogEC_{50}$	5.9±0.1	n.d.	5.9±0.9	n.d.	n.d.	5.6±0.7
1 nM	100.8±0.6	-	100.0±0	99.7±2.0	-	108.2±5.9
3 nM	-	-	-	110.8±0	-	-
10 nM	102.8±1.4	103.2±3.2	101.0±1	107.3±3.4	89.6±3.1	120.2±7.1
30 nM	-	-	-	113.5±0	-	-
100 nM	107.0±2.5	103.2±3.2	108±3.7	122.3±4.6	83.6±4.2	148.2±8.0
300 nM	120.5±4.3	-	104±4.0	-	-	-
1 µM	145.3±3.7	106±2.2	132.8±4.0	137±6.7	90.6±5.1	249.5±18.9
3 µM	196.7±13.7	-	-	-	-	-
10 µM	231.6±7.5	124±4.1	201.8±23	104±12.6	52.8±6.7	393.7±35.0
30 µM	137.3±17.8	-	195±0	-	-	-
n	3	4-6	4	7	4	4

EC_{50} (bold-faced) and efficacy of compound **3** (LAU206) at increasing concentrations in αkβ3γ2 (k=1-6) receptors. Compound **3** has low modulatory effects in α1- and α3β3γ2 receptors with an EC_{50} at ~1 µM. Due to the relatively low modulation in α2-, α4- and α5β3γ2 EC_{50} values could not be obtained. Table 1 was prepared from this data. Control current = 100% (GABA $EC_{3\text{-}5}$). (n.d.=not determined)

Supplementary Table S16:

LAU156 (**2**)	α1β3	α2β3	α3β3	α5β3
**EC_{50} [µM]**	**2.4**	**3.7**	**2.1**	**6.1**
$LogEC_{50}$	-5.6±0.1	-4.8±0.8	-5.7±0.04	-5.2±0.7
1 nM	-	100.0±0	101.2±0.8	97.3±1.2
3 nM	100.1±0.1	-	-	-
10 nM	100.7±0.5	100.0±0	102.4±1.2	105.5±2.8
30 nM	-	-	-	-
100 nM	109.0±2.2	108.2±3.0	105.9±1.3	109.5±3.6
300 nM	122.5±5.4	122.8±4.9	119.9±2.4	108.1±6.3
1 µM	151.2±11.5	126.9±9.8	150.6±2.2	132.0±3.8
3 µM	276.2±41.7	253.5±4	226.2±10.7	190.3±22.9
10 µM	372.3±40.1	254.0±31.9	284.8±7.0	189.7±18.1
30 µM	374.9±34.7	359.4±18.2	288.1±8.7	231.9±21.5
n	6-8	3-6	4	3-7

EC_{50} (bold-faced) and efficacy of compound **2** (LAU156) at increasing concentrations in αkβ3 (k=1,2,3 and 5) receptors. Control current = 100% (GABA $EC_{3\text{-}5}$).

Supplementary Table S17:

LAU206 (**3**)	α1β3	α2β3	α3β3	α5β3
**EC_{50} [μM]**	**2.2**	**5.1**	**1.9**	**n.d.**
$LogEC_{50}$	-5.7±0.1	-5.3±2.6	-5.7±0.8	n.d.
1 nM	108.1±1.8	100.0±0.1	103.0±3.1	-
3 nM	-	-	-	-
10 nM	105.0±4.0	101.0±0.5	112.6±2.7	105.2±2.6
30 nM	-	-	-	-
100 nM	117.8±5.9	101.8±1.2	116.4±3.0	115.4±8.0
300 nM	128.1±8.0	-	-	-
1 μM	162.2±15.9	113.9±2.0	144.4±6.3	107.7±4.7
3 μM	254.1±28.7	-	-	-
10 μM	313.7±13.6	156.4±9.7	175.5±6.8	75.4±6.4
30 μM	-	-	-	-
n	7-8	4-6	4	4

EC_{50} (bold-faced) and efficacy of compound **3** (LAU206) at increasing concentrations in αkβ3 (k=1,2,3 and 5) receptors. Due to the relatively low modulation in α5β3γ2 EC_{50} values could not be obtained. Control current = 100% (GABA EC_{3-5}). (n.d.=not determined).

Supplementary Table S18:

PZ II 028	α1β1γ1	α1β1δ
**EC_{50} [μM]**	**0.08**	**0.07**
$LogEC_{50}$	-7.0 ±0.1	-7.0±0.2
nH	1.1±0.4	0.8±0.2
1 nM	109.9±9.9	103.7±1.6
3 nM	-	122.9±3.9
10 nM	114.2±9.9	126.3±5.6
30 nM	-	191.7±21.1
100 nM	216.6±9.7	213.4±12.8
300 nM	-	331.9±3.2
1 μM	320.5±17.9	312.4±16.9
3 μM	-	367.7±10.8
10 μM	300.4±20.8	294.5±11.1
30 μM	-	-
n	3	3-10

EC_{50} (bold-faced), Hill slope (nH) and efficacy of compound **1** (PZ II 028) at increasing concentrations in α1β1γ1 and α1β1δ receptors, compare to EC_{50} = 0.13 μM in the α1β1 receptor (Supplementary Table S1). Figure 5 was prepared from this data, which is reported as mean ± SEM. Control current = 100% (GABA EC_{3-5}).

Supplementary Table S19:

LAU462 (**7**)	α1β1γ1	α1β1δ
**EC_{50} [μM]**	**21**	**2.3**
$LogEC_{50}$	4.7±1.4	5.6±0.5
nH	0.6±0.2	1.1±0.8
1 nM	102.6±0.4	100.6±0.6
3 nM	105.1±0.7	100.0±0.7
10 nM	106.0±1.9	105.5±2.8
30 nM	107.7±1.4	108.0±5.2
100 nM	112.0±0.9	128.1±3.4
300 nM	114.9±2.0	163.0±6.2
1 μM	120.8±3.2	380.9±48.7
3 μM	158.7±3.2	636.0±116.0
10 μM	175.1±13.6	885.3±293.0
30 μM	-	-
n	3	3-4

EC_{50} (bold-faced), Hill slope (nH) and efficacy of compound **7** (LAU462) at increasing concentrations in α1β1γ1 and α1β1δ receptors. Figure 6 was prepared from this data, which is reported as mean ± SEM. Control current = 100% (GABA EC_{3-5}).

Supplementary Figure S20:

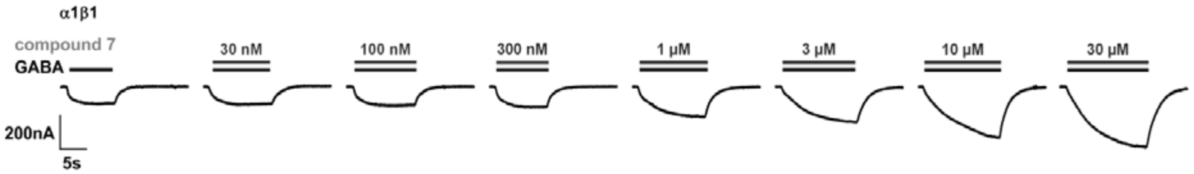

Original current traces showing the potentiation of the GABA-elicited response (EC_{3-5}) by increasing concentrations of compound **7** in the α1β1 receptor. For this compound we note that current rise is slowed even more compared to the other six ligands.

Synthesis of precursors

Commercially available reagents were used without further purification. Reactions were monitored by thin layer chromatography with silica gel 60 F_{254} plates (E. Merck, Darmstadt, Germany). HPLC chromatography was carried out with the Autopurification system by Waters using fluoro-phenyl columns. ^{1}H and ^{13}C NMR spectra were recorded on Bruker *AC 200* (^{1}H: 200MHz, ^{13}C: 50 MHz), Bruker *Avance Ultrashield 400* (^{1}H: 400 MHz, ^{13}C: 101 MHz) or Bruker *Avance IIIHD 600* spectrometer equipped with a Prodigy BBO cryo probe (^{1}H: 600 MHz, ^{13}C: 151MHz). Chemical shifts are reported in parts per million (ppm) and were calibrated using DMSO-d_6 as internal standard. Multiplicities are denoted by s (singlet), br s (broad singlet), d (doublet), dd (doublet of doublet) and m (multiplet). Melting points were determined with a Büchi Melting Point B-545 apparatus. HR-MS was measured on an Aglient 6230 LC TOFMS mass spectrometer equipped with an Aglient Dual AJS ESI-Source. Spectra of literature unknown compounds are shown in Supplementary Figures S25-S32.

Precursor for the synthesis of **6** (DCBS96):

8-Methoxy-2-(4-nitrophenyl)-2,5-dihydro-3H-pyrazolo[4,3-c]quinolin-3-one

Ethyl 4-chloro-6-methoxyquinoline-3-carboxylalte (63 mg, 0.24 mmol, 1 eq.) and the 4-nitrophenylhydrazine hydrochloride (67 mg, 0.36 mmol, 1.5 eq.) were dispersed in 3 mL diphenylether, Et_3N (0.62 mmol, 2.6 eq.) was added and the reaction mixture was heated to 150 °C under argon atmosphere. After 20 h the reaction mixture was cooled to room temperature and was rinsed with 3 mL of a 1/1 mixture of petroleum ether (PE) and ethyl acetate (EtOAc). The precipitate was collected by filtration, washed with 15 mL water and 30 mL PE/EtOAc (1/1) to give 8-methoxy-2-(4-nitrophenyl)-2,5-dihydro-3*H*-pyrazolo[4,3-c]quinolin-3-one as orange solid (50 mg, 0.15 mmol, 63%). ^{1}H NMR (600 MHz, DMSO-d_6) δ 3.94 (s, 3H), 7.34 (dd, *J* = 9.0, 2.9 Hz, 1H), 7.60 (d, *J* = 2.8 Hz, 1H), 7.70 (d, *J* = 9.1 Hz, 1H), 8.31 – 8.38 (m, 2H), 8.52 – 8.57 (m, 2H), 8.77 (d, *J* = 6.6 Hz, 1H), 13.04 (br d, *J* = 6.6 Hz, 1H). ^{13}C NMR (151 MHz, DMSO-d_6) δ 55.8, 102.7, 104.5, 117.8 (2C), 119.8, 120.4, 121.5, 125.0 (2C), 130.1, 138.8, 142.5, 144.8, 145.2, 157.8, 162.8. M.p.: decomposes > 300 °C.

Precursor for the synthesis of **7** (LAU462):

N-(2-(*tert*-Butyl)phenyl)acetamide

2-*tert*-Butylaniline (8.10 g, 54 mmol) was dissolved in anhydrous pyridine (155 mL) and cooled to 0°C. Acetic anhydride (11.01 g, 108 mmol) was added dropwise. The reaction was allowed to warm to room temperature and stirred for 6 h. The solvent was evaporated under reduced pressure and the residue was recrystallized from MTBE (1000 mL) to give the N-(2-(*tert*-butyl)phenyl)acetamide as colorless needles (8.60 g, 45 mmol, 83%). ^{1}H NMR (400

MHz, DMSO-d_6) δ 1.32 (s, 9H), 2.03 (s, 3H), 6.98 – 7.06 (m, 1H), 7.14 – 7.24 (m, 2H), 7.35 – 7.42 (m, 1H), 9.20 (s, 1H). ^{13}C NMR (101 MHz, DMSO-d_6) δ 23.1, 30.7, 34.7, 126.2, 126.4, 126.7, 131.8, 136.1, 146.3, 169.0. TLC (PE/EtOAc = 4/1): R_f=0.21. M.p.: 164 - 165°C.

N-(2-(*tert*-butyl)-4-chlorophenyl)acetamide

N-(2-(*tert*-butyl)phenyl)acetamide (8.35 g, 44 mmol) and potassium chloride (4.65 g, 62.4 mmol) were suspended in acetonitrile (210 mL). Oxone (19.19 g, 62.4 mmol) was added in small portions. The reaction mixture was stirred at room temperature. After 24 h the reaction mixture was filtered and evaporated. The pure product was obtained after recrystallization of the residue from toluene/Et_2O (1/1) to give the N-(2-(*tert*-butyl)phenyl)acetamide as colorless solid (4.24 g, 19.4 mmol, 44%). ^{1}H NMR (400 MHz, DMSO-d_6) δ 1.31 (s, 9H), 2.03 (s, 3H), 7.06 (d, *J* =8.3 Hz, 1H), 7.26 (dd, *J* = 8.3 Hz, 2.3 Hz, 1H), 7.35 (d, *J* = 2.3 Hz, 1H), 9.25 (s, 1H). ^{13}C NMR (101 MHz, DMSO-d_6) δ 23.1, 30.3, 34.9, 126.2, 126.5, 131.0, 133.6, 135.1, 148.7, 169.2. TLC (PE/EtOAc = 3/2): R_f=0.53. M.p.: 128 - 131°C.

2-(*tert*-butyl)-4-chloroaniline

N-(2-(*tert*-butyl)-4-chlorophenyl)acetamide (4.24 g, 18.8 mmol) was heated to reflux in 7 M HCl for 18 h. The reaction was basified with 2M NaOH and extracted three times with Et_2O. The combined organic layers were washed with brine, dried over Na_2SO_4 and evaporated to give the desired 2-(*tert*-butyl)-4-chloroaniline as brown oil (3.33 g, 14.9 mmol, 79%). ^{1}H NMR (400 MHz, $CDCl_3$) δ 1.40 (s, 9H), 3.81 (s, 2H), 6.56 (d, *J* = 8.4 Hz, 1H), 6.98 (dd, *J* = 8.4, 2.4 Hz, 1H), 7.18 (d, *J* = 2.4 Hz, 1H). ^{13}C NMR (101 MHz, $CDCl_3$) δ 29.4, 34.5, 118.9, 123.4, 126.7, 126.8, 135.5, 143.3. TLC (PE/EtOAc = 4/1): R_f=0.64.

Diethyl 2-(((2-(*tert*-butyl)-4-chlorophenyl)amino)methylen)malonate (see Supplementary Figures S25 and S26, THK17)

Diethyl 2-(((2-(*tert*-butyl)-4-chlorophenyl)amino)methylen)malonate was synthesized according to the literature[2-4] in 95% yield (colorless solid, 5.52 g, 15.7 mmol). ^{1}H NMR (200 MHz, DMSO-d_6) δ 1.23 (t, *J* = 7.1 Hz, 3H), 1.25 (t, *J* = 7.1 Hz, 3H), 1.39 (s, 9H), 4.11 (q, *J* = 7.1, 6.5 Hz, 2H), 4.22 (q, *J* = 6.5, 6.0 Hz, 2H), 7.20 – 7.49 (m, 3H), 8.30 (d, *J* = 13.3 Hz, 1H), 11.18 (d, *J* = 13.3 Hz). ^{13}C NMR (50 MHz, $CDCl_3$) δ 14.4, 14.5, 30.2, 34.8, 60.2, 60.6, 94.2, 122.5, 127.5, 127.5, 131.4, 137.4, 143.0, 154.2, 165.9, 169.2. HR-MS: calculated [$C_{18}H_{25}ClNO_4^+$]: 354.1473; found [$C_{18}H_{25}ClNO_4^+$]: 354.1484 (diff.: 5.16 ppm). TLC (PE/EtOAc = 4/1): R_f=0.83. M.p.: 70-71°C.

Ethyl 8-(*tert*-butyl)-6-chloro-4-oxo-1,4-dihydroquinoline-3-carboxylate (see Supplementary Figures S27 and S28, THK20)

Ethyl 8-(*tert*-butyl)-6-chloro-4-oxo-1,4-dihydroquinoline-3-carboxylate was synthesized according to the literature[2-4] in 77% yield (colorless solid, 268 mg, 0.88 mmol). ^{1}H NMR (200 MHz, $CDCl_3$) δ 1.47 (t, *J* = 7.1 Hz, 3H), 1.63 (s, 9H), 4.50 (q, *J* = 7.1 Hz, 2H), 7.66 (d, *J* = 2.4 Hz, 1H), 8.19 (d, *J* = 2.4 Hz, 1H), 9.13 (s, 1H), 12.10 (s, 1H). ^{13}C NMR (50 MHz, $CDCl_3$) δ 14.2, 30.7, 36.8, 61.9, 103.4, 120.4, 121.6, 130.1, 131.8, 147.0, 148.5, 150.4, 165.8, 170.2. HR-MS: calculated [$C_{16}H_{19}ClNO_3^+$]: 308.1055; found [$C_{16}H_{19}ClNO_3^+$]: 308.1053 (diff.: 2.58 ppm). TLC (PE/EtOAc = 30/1): R_f=0.44. M.p.: 138-139°C.

Ethyl 8-(*tert*-butyl)-4,6-dichloroquinoline-3-carboxylate (see Supplementary Figures S29 and S30, THK32)

Ethyl 8-(*tert*-butyl)-4,6-dichloroquinoline-3-carboxylate was synthesized according to the literature[2-4] in yields of 91% (off white solid, 495 mg, 1.52 mmol). ^{1}H NMR (400 MHz, $CDCl_3$) δ 1.46 (t, *J* = 7.1 Hz, 3H), 1.64 (s, 9H), 4.50 (q, *J* = 7.1 Hz, 2H), 7.71 (d, *J* = 2.3 Hz, 1H), 8.31 (d, *J* = 2.3 Hz, 1H), 9.18 (s, 1H). ^{13}C NMR (101 MHz, $CDCl_3$) δ 14.2, 30.8, 37.0, 62.1, 122.6, 122.8, 127.9, 129.8, 134.2, 142.2, 147.0, 147.2, 151.1, 164.5. HR-MS: calculated [$C_{16}H_{18}Cl_2NO_2^+$]: 326.0716; found [$C_{16}H_{18}Cl_2NO_2^+$]: 326.0726 (diff.: 4.85 ppm). TLC (PE/EtOAc = 20/1): R_f=0.59. M.p.: 124°C.

Supplementary Figure S21: ^{1}H NMR of **5** (DCBS76)

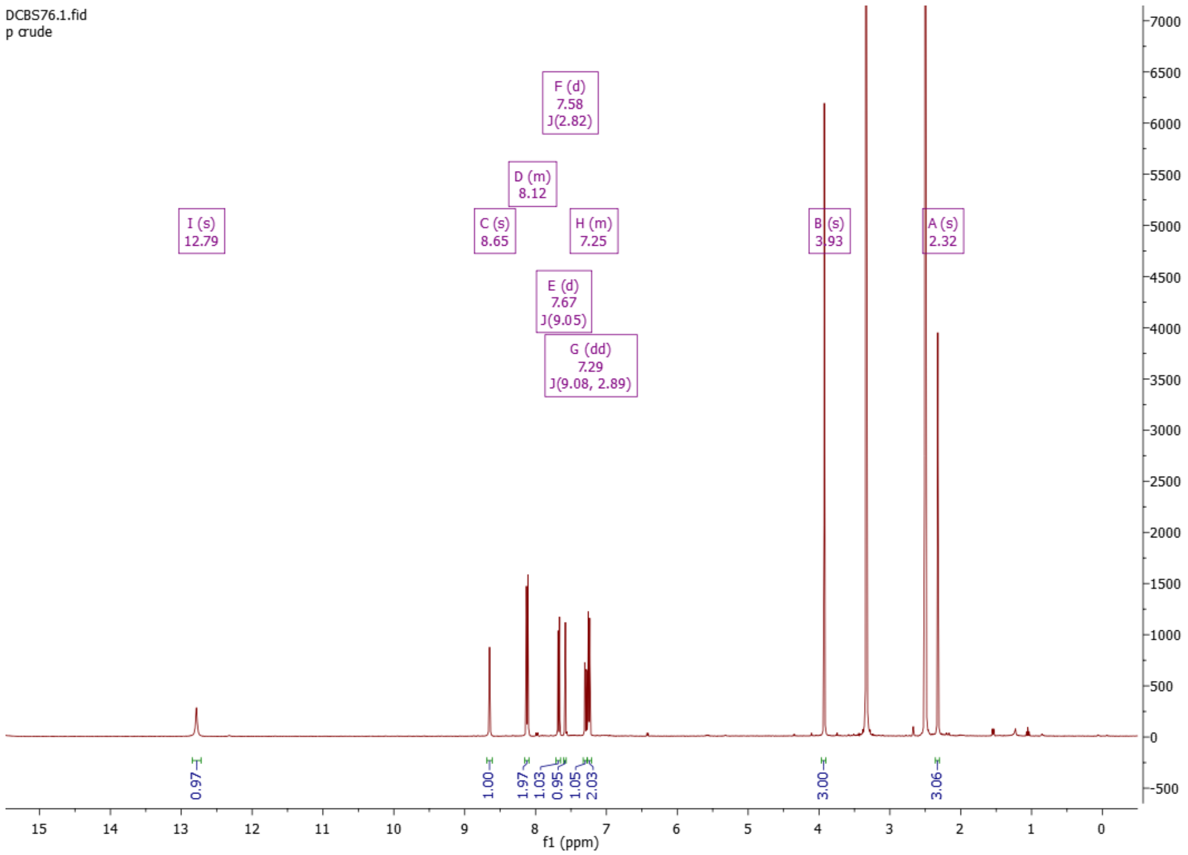

Supplementary Figure S22: ^{13}C NMR of **5** (DCBS76)

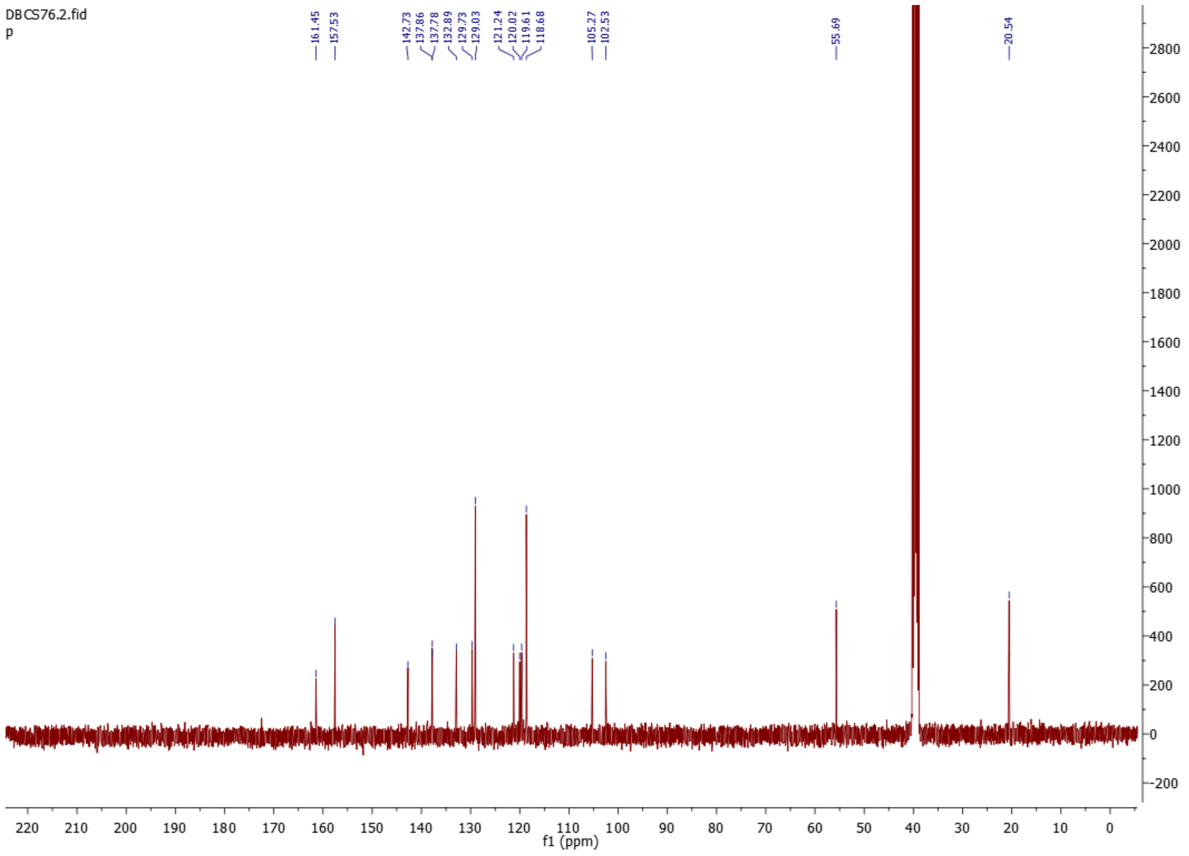

Supplementary Figure S23: ^{1}H NMR of **6** (DCBS96)

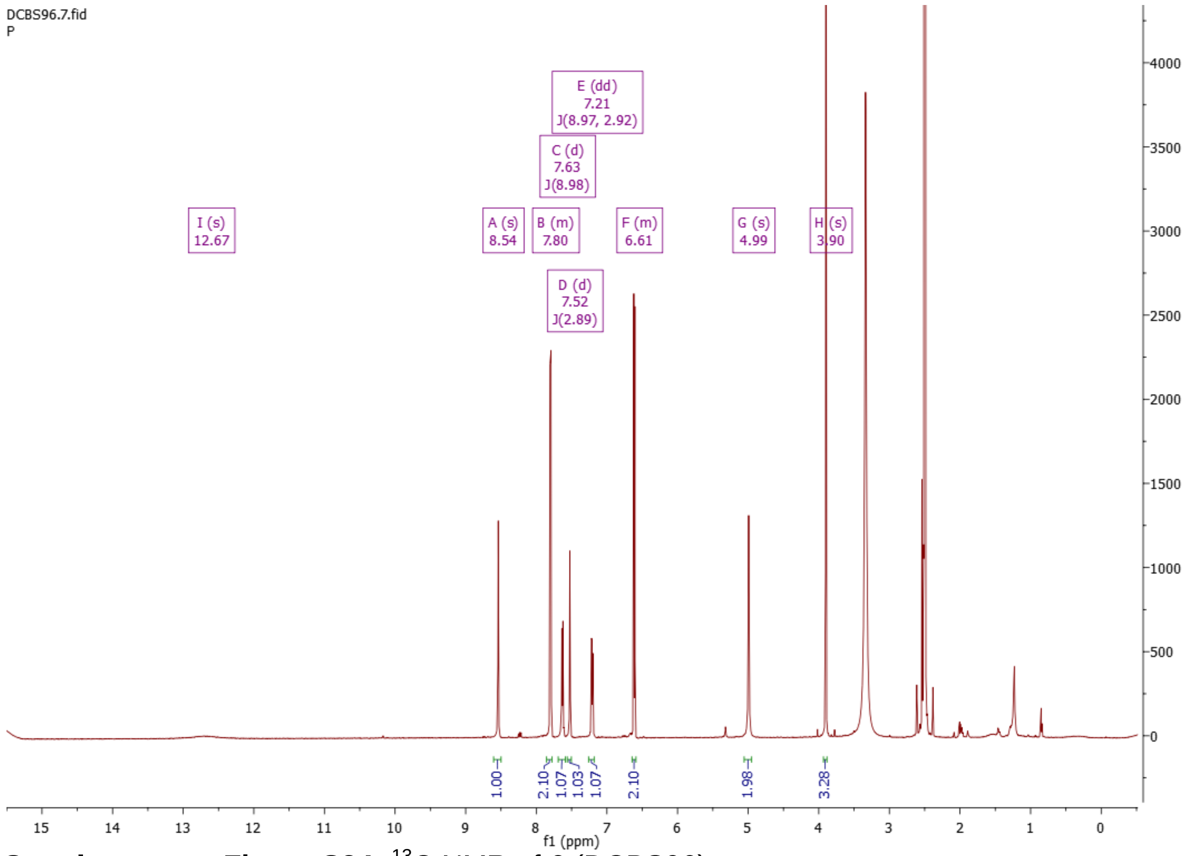

Supplementary Figure S24: ^{13}C NMR of **6** (DCBS96)

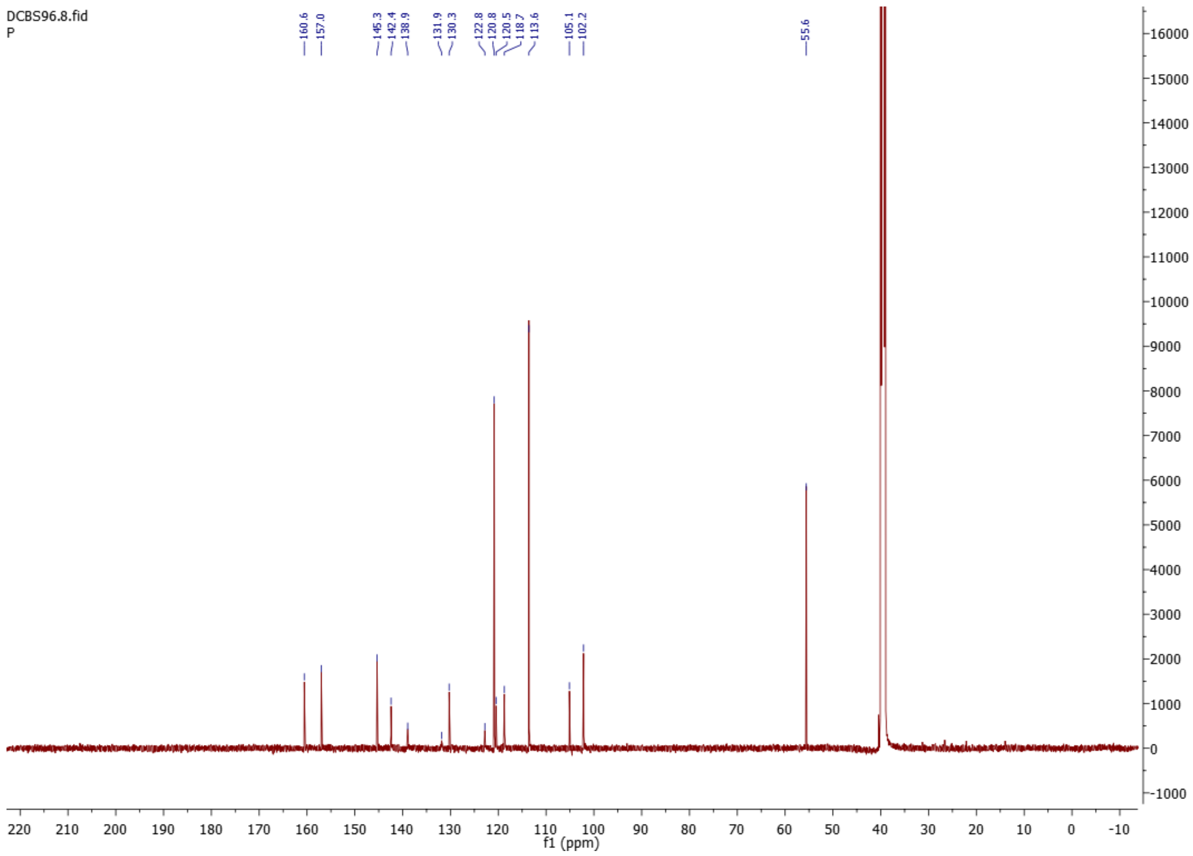

Supplementary Figure S25: ^{1}H NMR of THK17

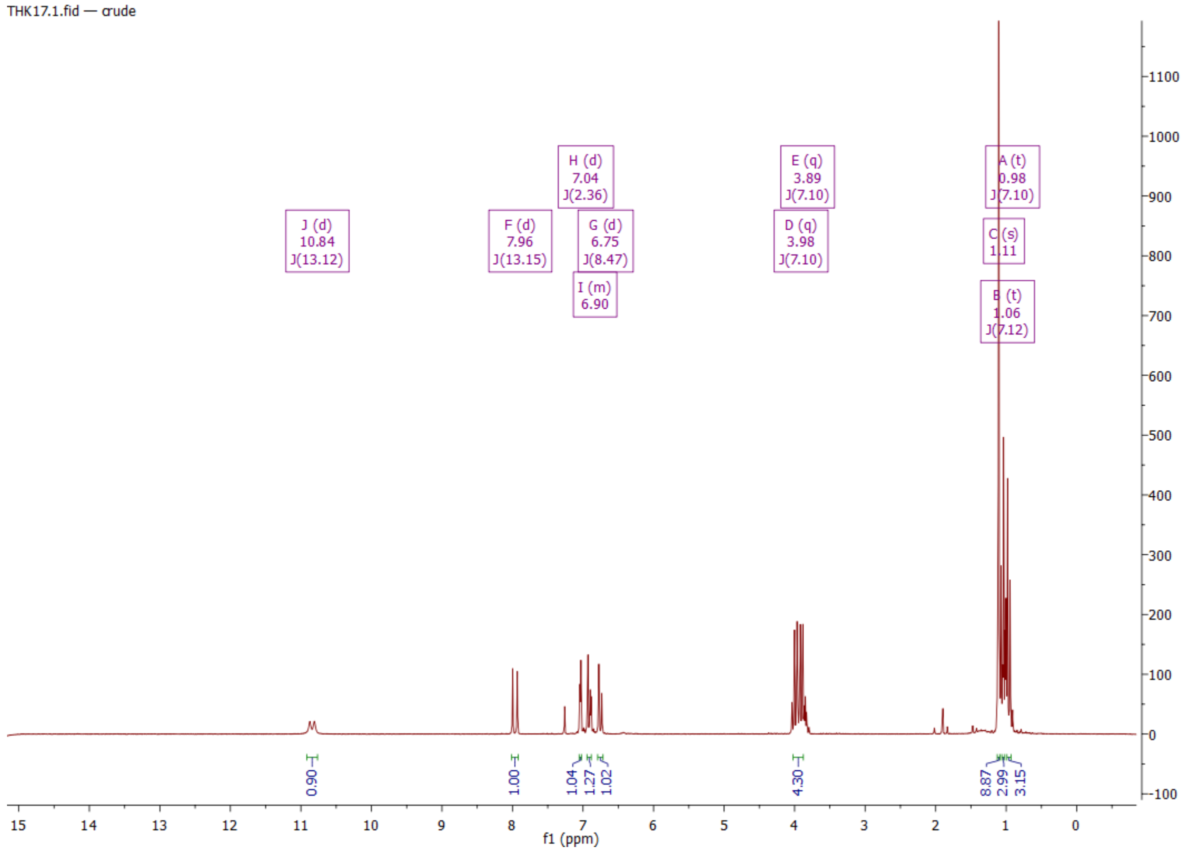

Supplementary Figure S26: APT NMR of THK17

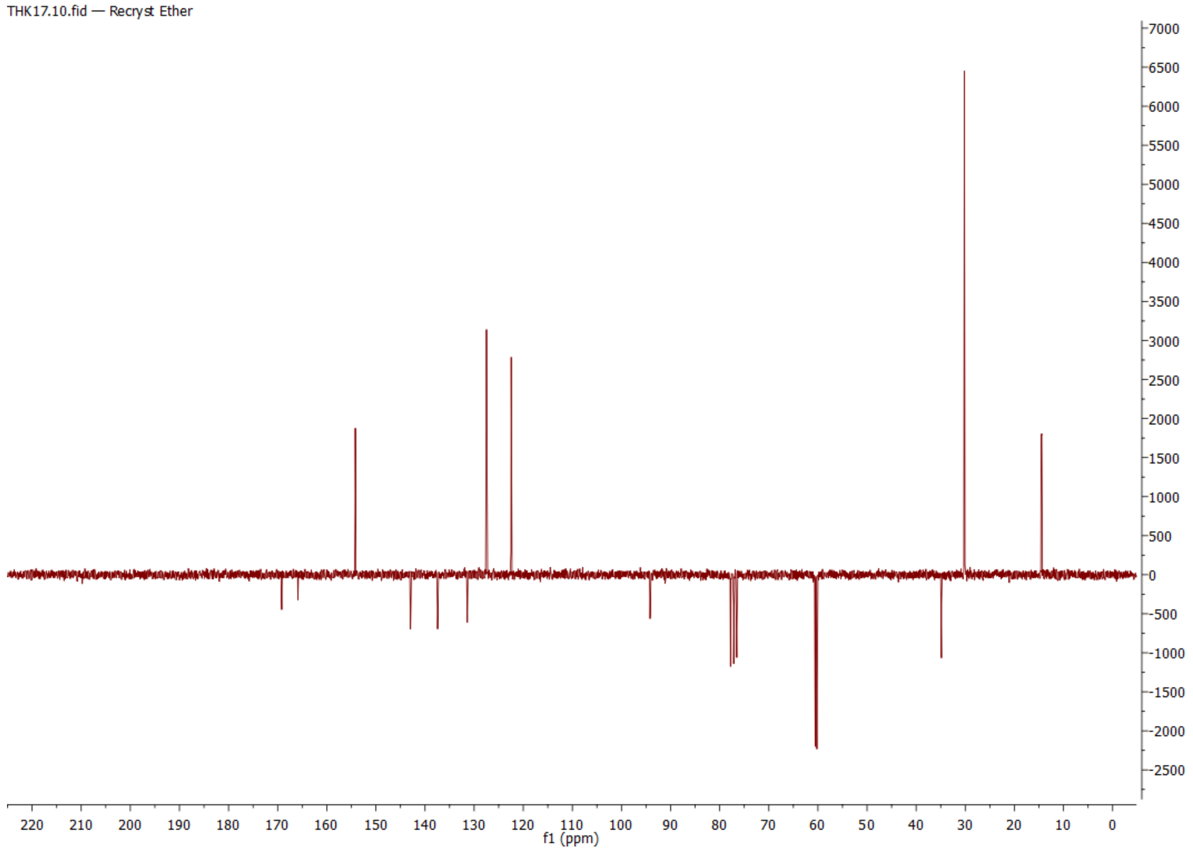

Supplementary Figure S27: ^{1}H NMR of THK20

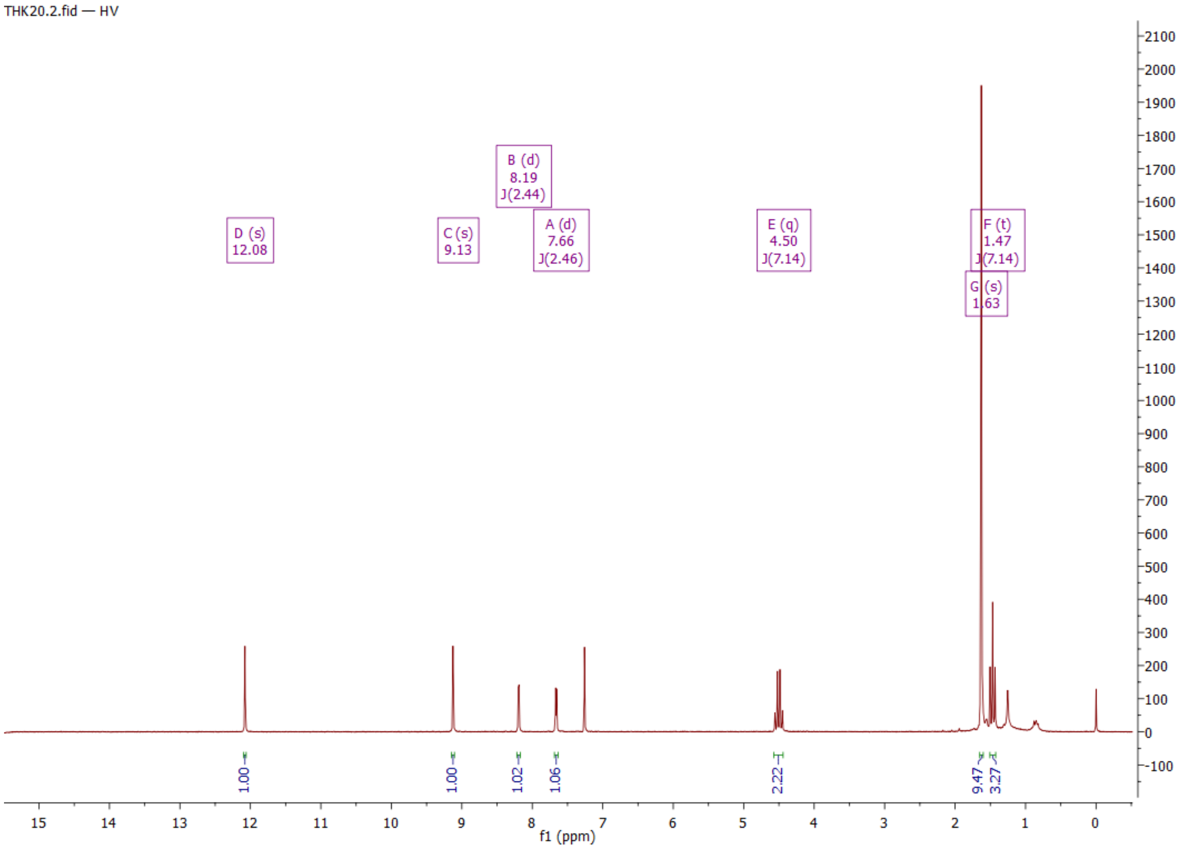

Supplementary Figure S28: APT NMR of THK20

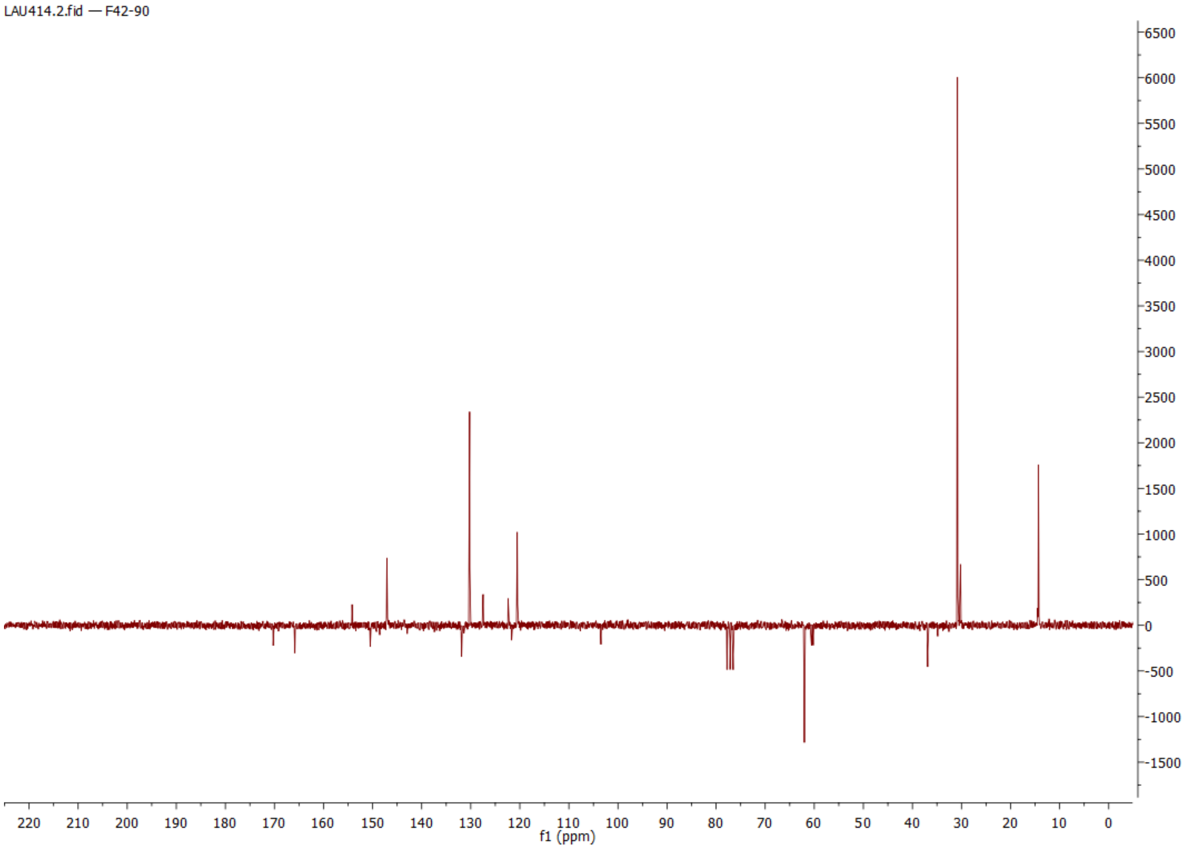

Supplementary Figure S29: ^{1}H NMR of THK32

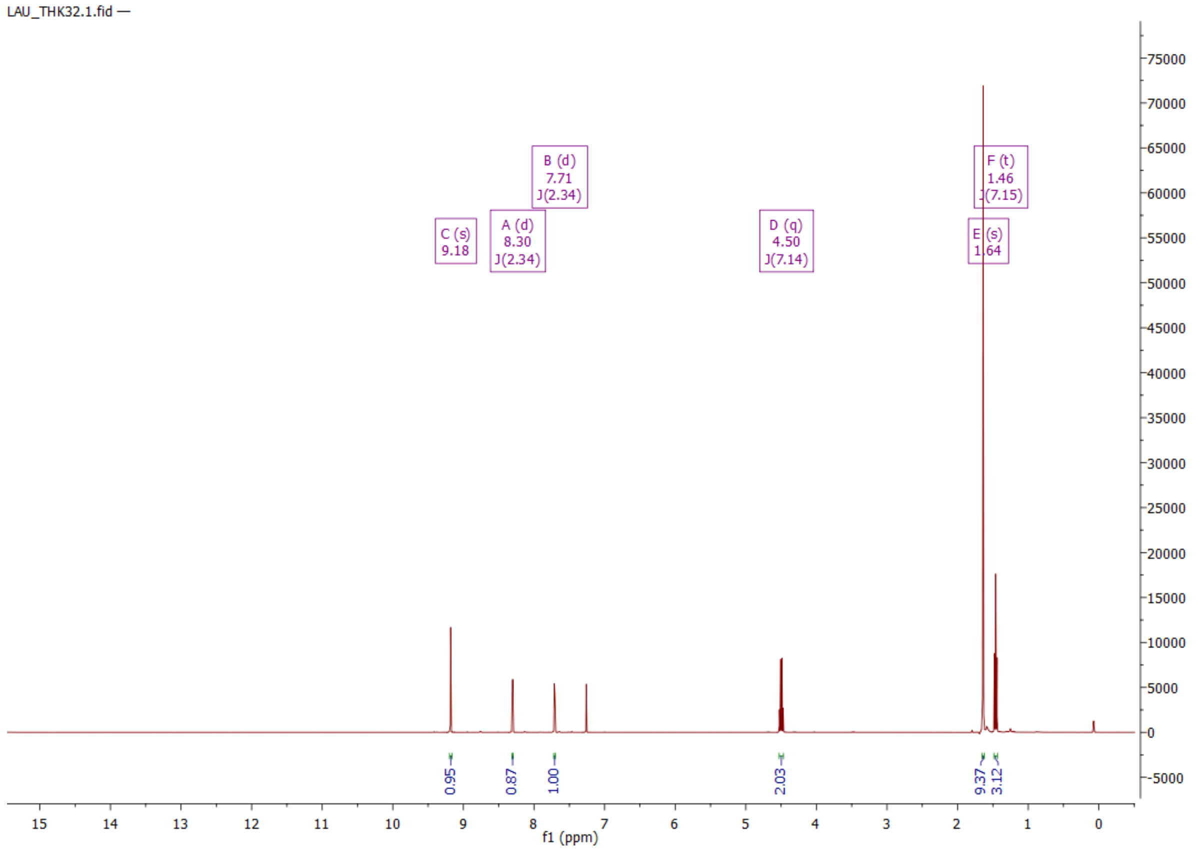

Supplementary Figure S30: ^{13}C NMR of THK32

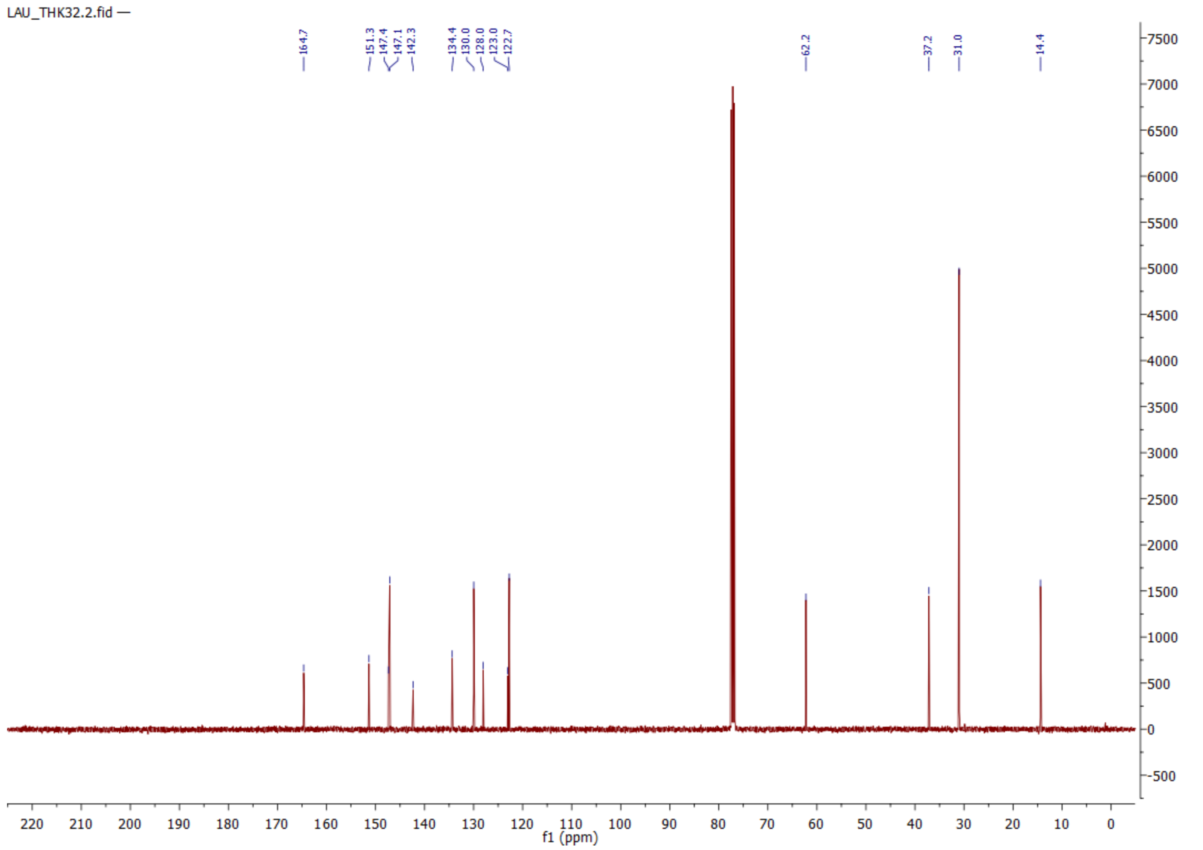

Supplementary Figure S31: ^{1}H NMR of **7** (LAU462)

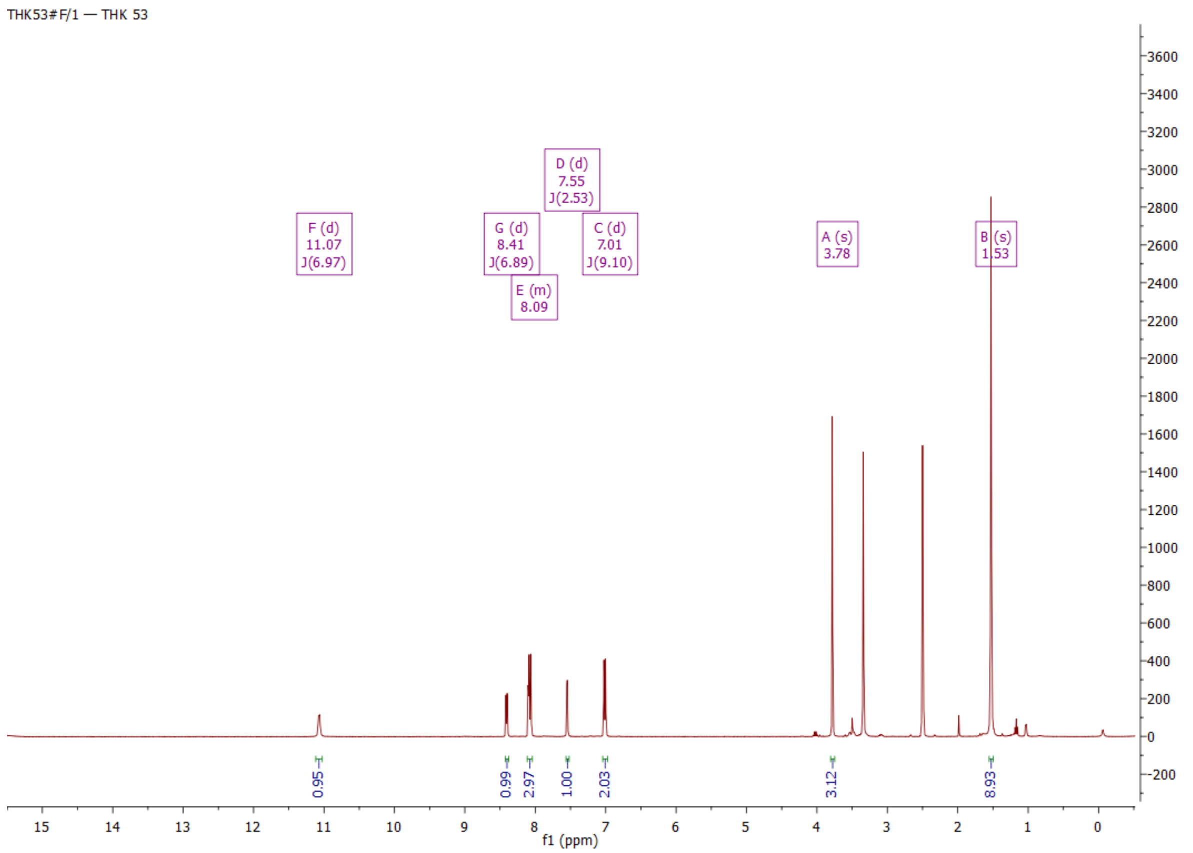

Supplementary Figure S32: ^{13}C NMR of **7** (LAU462)

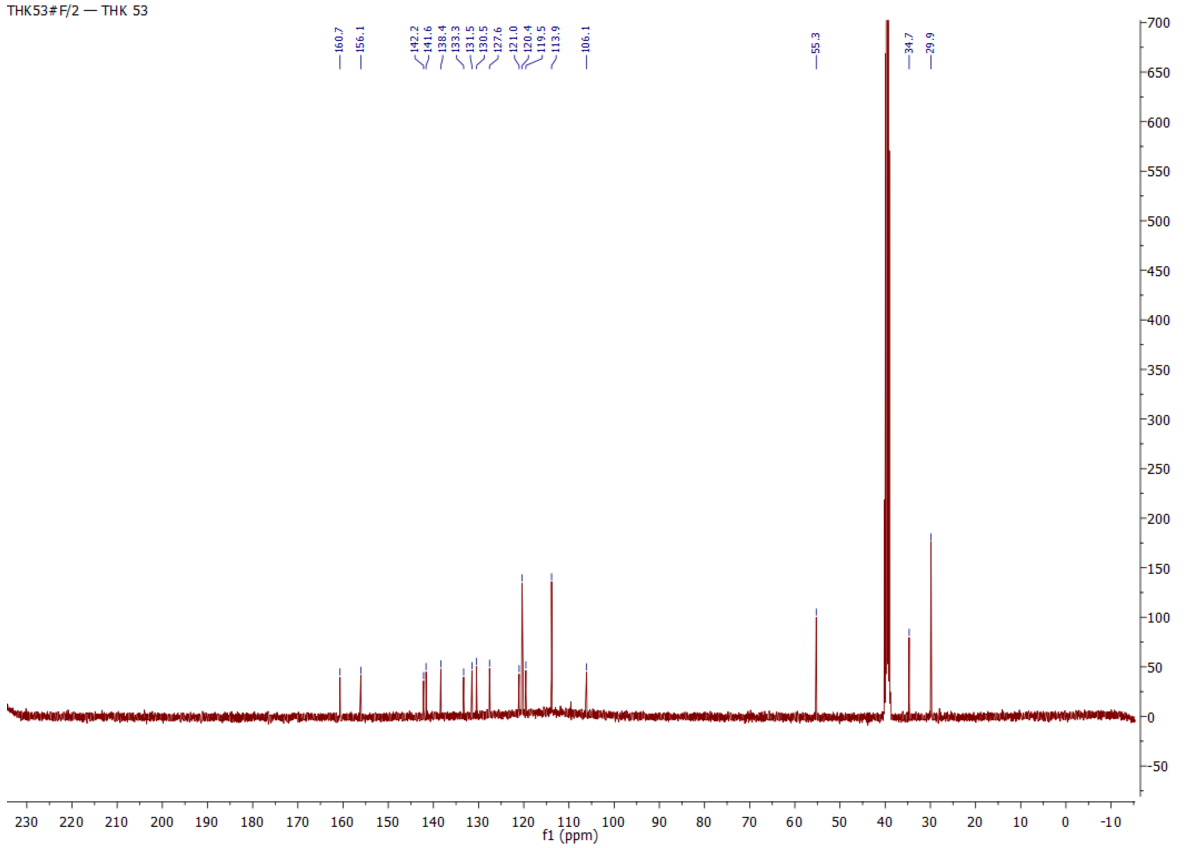

Supplementary References

1 Varagic, Z. *et al.* Subtype selectivity of alpha+beta- site ligands of $GABA_A$ receptors: identification of the first highly specific positive modulators at alpha6beta2/3gamma2 receptors. *Brit J Pharmacol* **169**, 384-399 (2013).

2 Fryer, R. I. *et al.* Structure-activity relationship studies at benzodiazepine receptor (BZR): a comparison of the substituent effects of pyrazoloquinolinone analogs. *J Med Chem* **36**, 1669-1673 (1993).

3 Hörlein, G., Kübel, B., Studeneer, A. & Salbeck, G. Heterocyclen durch Anellierung an 4-Pyridinole, II Thieno[3,2-c]pyridin-3-ole. *Liebigs Ann Chem* **1979**, 387-391 (1979).

4 Savini, L. *et al.* High affinity central benzodiazepine receptor ligands. Part 2: quantitative structure-activity relationships and comparative molecular field analysis of pyrazolo[4,3-c]quinolin-3-ones. *Bioorg Med Chem* **9**, 431-444 (2001).
